# From Waste to Function: Valorization of Collagen-Based Wastes with Natural Deep Eutectic Solvents for Bioadhesive Applications

**DOI:** 10.1021/acssuschemeng.5c11526

**Published:** 2026-02-03

**Authors:** Chiara Pelosi, Eleonora Micheli, Elena Pulidori, Giulia Caroti, Brunella Cipolletta, Beatrice Campanella, Iacopo Corsi, Silvia Pizzimenti, Leila Birolo, Ilaria Bonaduce, Celia Duce, Emilia Bramanti

**Affiliations:** † Department of Chemistry and Industrial Chemistry, 154932 University of Pisa, Via G. Moruzzi 13, 56124 Pisa, Italy; ‡ Institute of Chemistry of Organometallic Compounds, 117064 National Research Council, Via G. Moruzzi 1, 56124 Pisa, Italy; § Department of Chemical Science, University of Naples Federico II, Strada Comunale Cinthia 26, 80126, Napoli, Italy; ⊥ Conceria Zabri S.p.A., 50054, Fucecchio, Italy

**Keywords:** valorization of leather wastes, vegetable-tanned leather, Natural Deep Eutectic solvents (NADES), Adhesive biomaterials, Collagen

## Abstract

In this study, we propose a novel, environmentally, economically, and energetically sustainable approach for the valorization of vegetable-tanned leather waste, aiming at producing biobased adhesives with antibacterial properties and promising water resistance. The treatment of leather scraps was carried out using various Natural Deep Eutectic Solvents (NADES) in mild conditions (1 h, 60 °C), i.e., lactic acid:urea in a molar ratio of 2:1, choline chloride:lactic acid in a molar ratio of 1:1, and choline chloride dehydrated oxalic acid in a molar ratio of 1:1. The resulting bioadhesives exhibited excellent binding performances, in particular, on wood substrates. Structural modifications and its thermal behavior of collagen after the treatment were investigated using Fourier Transform Infrared Spectroscopy with Attenuated Total Reflectance (ATR-FTIR), Thermogravimetric Analysis (TGA and TGA/FTIR), Evolved Gas Analysis–Mass Spectrometry (EGA-MS), Pyrolysis–Gas Chromatography–Mass Spectrometry (Py-GC-MS), and proteomic techniques. Overall, this approach highlights a circular and green strategy for upcycling leather industry byproducts into high-performance materials, aligning with current goals in waste minimization and resource efficiency.

## Graphical abstract

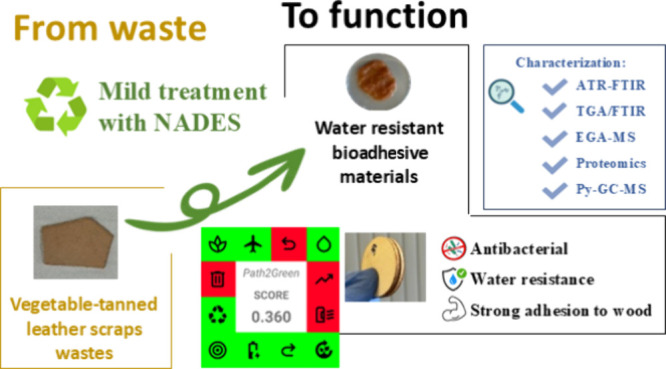

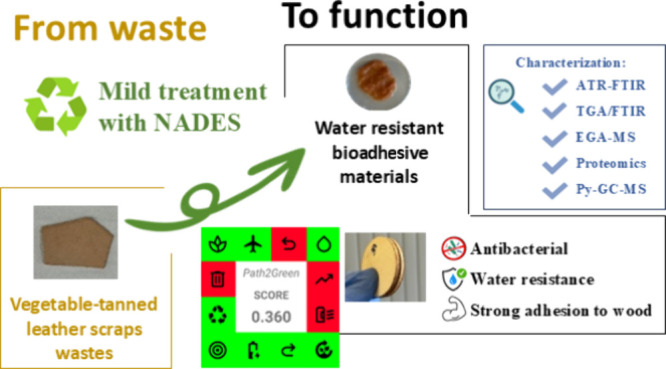

## 1 Introduction

Leather, traditionally produced processing animal hides, is recognized as one of the earliest materials utilized by humans.^1^ However, its production (which currently has an estimated annual revenue of USD 50 billion) poses significant environmental challenges, including the management of the solid waste generated.^2^ Collagen, a fibrillar protein, is abundantly present in the solid wastes.^2,3^ Collagen can be found in several forms; 29 distinct types have been identified so far. Type-I collagen (composed of three polypeptide chains with a triple helical structure) is the most abundant form. The helices are characterized by repeating Gly-X-Y sequences, where the X and Y positions are mainly proline and hydroxyproline residues, respectively.^4,5^

Numerous studies in the literature highlight the valorization of collagen for applications across a wide range of fields, including food, pharmaceutical, cosmetic, or biomedical industries.^4,6–9^ Despite this potential, the recovery and reuse of collagen from leather industry residues are complicated by the fact that the biomacromolecule has previously undergone a tanning process, resulting in its stabilization through cross-linking. Different tanning methods are available on the market, depending on the quality of the leather required by consumers, mainly divided into mineral tanning (made with chromium salts, which is the most common, or aluminum, iron, or zinc salts) and organic tanning (including vegetable, aldehyde, or oil tanning^10,11^ ). The vegetable tanning is based on the formation of multihydrogen links between the polyphenols extracted from different vegetable matrices (e.g., chestnut, mimosa, and quebracho tree) and collagen, making the latter nonbiodegradable and resistant to high temperature.^12^

While the recovery of collagen from chromium-tanned leather is particularly challenging due to the strong coordination bonds formed with chromium­(III), an interesting approach, so far unexplored in the scientific literature, is the sustainable valorization of solid wastes derived from the vegetable-tanning industry. In this article, we present an innovative green treatment of vegetable-tanned leather waste using three different Natural Deep Eutectic Solvents (NADESs), leading to the formation of bioadhesives.

Deep eutectic solvents (DESs) constitute a class of fluids that have garnered significant attention due to their wide-ranging potential applications in various technological fields, e.g., materials development,^13^ gas storage,^14,15^ polymer science,^16,17^ biomass extraction,^18–20^ electroplating,^21,22^ and separation techniques.^23^ They are usually prepared by combining two or more components forming an eutectic mixture whose melting point is lower than what would be expected if the mixture had an ideal behavior in the liquid phase.^24^ They are considered a platform of green solvents, whose properties can be adjusted according to the specific purpose, e.g., varying the starting components or with the addition of a cosolvent.^25,26^ Natural deep eutectic solvents (NADESs) are of particular interest, as they are formulated entirely from natural, biodegradable components. These solvents offer advantages such as low toxicity and reduced production costs, making them highly suitable for implementation within the framework of sustainable chemistry.^23^

In this study, we propose the use of (i) lactic acid:urea in a molar ratio of 2:1 (namely DES1); (ii) choline chloride:lactic acid in a molar ratio of 1:1 (namely DES2); (iii) choline chloride:dehydrated oxalic acid in a molar ratio of 1:1 (namely DES3), already classified in the literature as natural deep eutectic solvents (NADESs),^27–30^ to extract collagen from vegetable-tanned leather waste. The constitutes of the NADESs are cheap, biodegradable, and nontoxic, thus considered suitable for environment-friendly processes. Moreover, they have been recently proposed for collagen extraction from untanned fish skin, and for the solubilization of native collagen,^31–33^ making them promising for our purposes.

More in detail, Abdallah et al. have employed DESs based on urea (U) and lactic acid (LA) for the extraction of bioactive compounds from marine byproducts, which are typically discarded in large quantities.^31^ Similarly, Bisht et al. have utilized an aqueous 0.75 M solution of LA and U in a 2:1 molar ratio to extract type I collagen from the skin of Atlantic cod.^34^ The system has demonstrated high efficiency in extracting bioactive compounds from plant-based matrices under ultrasound-assisted conditions.^29^ Hamdi et al. developed a method for isolating type I collagen from seabass fish scales, preserving the native collagen’s secondary and triple helical structures, utilizing a ChCl:LA-based system-assisted ultrasonication (US) technical route.^35^ In another study, the authors monitored collagen solubilization at different temperatures (45 °C, 70 °C, and 90 °C), followed by regeneration at 4 °C using ethanol.^32^ DES2 has been applied to other valuable processes, including the extraction of lignin and polyphenols.^30,36^ ChCl:AO, i.e., the DES3 proposed in this study, has been reported in the literature for a variety of applications,^18,37–40^ including the extraction of approximately 50% high-purity collagen peptides from cod skin (ChCl:AO (1:1) at 45 °C).^33^

In the present paper, the treatment of vegetable tanned leather with DESs1–3 was conducted under mild experimental conditions (60 °C for 1 h), following the main principles of green chemistry: the biomass treated was a waste material furnished by a local industry, which was fully converted into a bioadhesive using green solvents. Only the leather treated with DES1 and DES2 produced bioadhesives with good adhesive properties. The resulting materials were successfully employed as biobased glue as-is or treated in an acidic or basic environment to explore the pH effect on their properties. The glue strength on steel-wood surfaces was also quantified by a tack test using a rheometer.

A unique characteristic of the bioadhesives proposed in this study is that, unlike the adhesives reported in the literature,^41–46^ they inherently contain tannins that enhance their moisture resistance^47^ making them particularly promising for bonding organic surfaces (such as paper-to-paper or wood-to-wood) under high-humidity conditions. Moreover, tannins are naturally occurring polyphenols that possess antimicrobial properties.^47–49^

To understand how treatment with NADESs affected collagen at the molecular level and how the protein chemical and structural changes influenced their adhesive properties, the biomaterials obtained were studied by Fourier-Transformed Infrared Spectroscopy (FTIR), Thermogravimetric Analysis (TGA) coupled with FTIR spectroscopy of evolved gases, Evolved Gas Analysis with Mass spectrometry (EGA-MS), pyrolysis–Gas Chromatography–Mass Spectrometry (Py-GC-MS), and Proteomic (LC-MSMS) analysis. The collagen extracted was compared with collagen present in the original vegetable-tanned leather to assess the molecular-level modifications induced by the NADES treatments.

Overall, the results obtained demonstrate how an innovative and green process, which employs NADESs, can valorize waste from the leather industry to produce biomaterials with adhesive properties, in line with the principles of the circular economy.

## 2 Materials and Methods

### 2.1 Materials

Vegetable-tanned calf split leather (containing tannins, e.g., mimosa, quebracho, and chestnut extract) was kindly provided by Conceria Zabri (Fucecchio, Italy). Two commercial animal glues, BM5 and HP3, were sourced from the Museo Nacional del Prado (Madrid) and from the restoration workshop of the University Suor Orsola Benincasa (Naples). Collagen from calf skin (CAS-No: 9007-34-5, purity 99.9%) was obtained from Sigma-Aldrich. Choline chloride (CAS-No: 67-48-1, ChCl, purity 98%) was purchased by Thermo Scientific and used as a component of the eutectic solvents after drying at approximately 100 °C under a vacuum for 2 h. Urea (CAS-No: 57-13-6, U, purity 99%) was purchased by ChemCruz. Lactic acid (CAS-No: 50-21-5, LA, purity 98%) was obtained by Carlo Erba. Sodium hydroxide (CAS-No: 1310-73-2, NaOH, ACS reagent, reag. Ph. Eur., ≥98%, pellets) and hydrochloric acid (CAS-No: 7647-01-0, HCl, ACS reagent, reag. ISO, reag. Ph. Eur., ≥37%) were purchased by Sigma-Aldrich. Dehydrated oxalic acid (CAS-No: 6153-56-6, OA. Purity 98%) was obtained by Materiamadre. Unless otherwise specified, chemicals were purchased and used without further purification.

### 2.2 Methods

#### 2.2.1 Preparation of Deep Eutectic Solvents (DESs)

The DES selected for the treatment of tanned leather is described in Table 1.

**Table 1:** Deep Eutectic Solvents Prepared in This Study

system name	components	molar ratios
DES1	Lactic acid:urea	2:1
DES2	Choline chloride:lactic acid	1:1
DES3	Choline chloride:dihydrated oxalic acid	1:1

All the systems were prepared by mixing the components at 50 °C and stirring them for 20 min (DES1) or 1 h (DES2 and DES3). Samples were stored at room temperature and used directly as solvents for the treatment of vegetable-tanned leather without any further processing.

#### 2.2.2 Mild Treatment of Vegetable-Tanned Leather Waste

For our purposes, vegetable-tanned leather was grated (Figure S1A–B) using a commercial grater with an approximate pore size of 0.5 mm, and then treated with DES1, DES2, and DES3 (Figure 1) using 100 mg of leather in 2 mL of DES. The mixture was kept at 60 °C for 1 h under magnetic stirring. Subsequently, 1 mL of water was added to the system and stirred at room temperature for another 30 min. After this step, samples were centrifuged (∼14000 rpm, at 25 °C for 5 min), and the solid residue—namely gelatin—was collected. The obtained gelatin was washed three times with water to remove most of the residual DES. The wet gelatin sample (Gel_x) was employed both without any further treatment or after treating it with 0.1 M HCl (Gel_x_HCl) or 0.1 M NaOH (Gel_x_NaOH) to evaluate the effect of pH on the protein structure. One mL of 0.1 M HCl or 0.1 M NaOH was added to the gelatin under magnetic stirring for 1 h at room temperature and centrifuged to remove the excess liquid.

**Figure 1:**
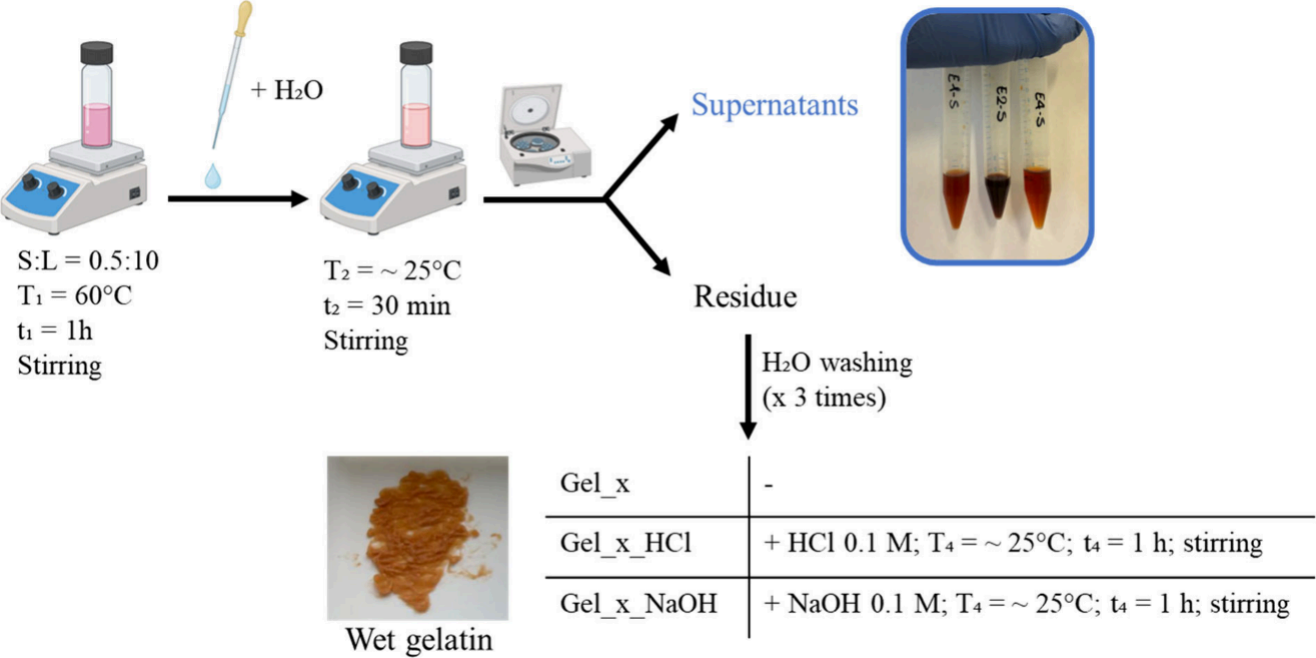
Scheme of the mild treatment of vegetable-tanned leather carried out with a solid-to-liquid ratio (S/L) of 0.5:10, where S refers to the vegetable-tanned leather and L refers to the deep eutectic solvent used (DES1, DES2, or DES3). The isolated supernatants and the gelatin obtained from the treatment (wet gelatin) are also shown.

To evaluate the recyclability of the solvents, all collected supernatants (from Gel_x tests) were left under a fume hood at room temperature for 24 h. These conditions proved to be effective in allowing a substantial portion of the water to evaporate. The regenerated DES was then subjected to a subsequent cycle of leather treatment, Gel_1_II° and Gel_2_II°, respectively.

Furthermore, to better understand the role of the DES in the treatment of tanned leather, a control experiment (CE) was performed using the same procedure but replacing the DES with water. Specifically, a solid-to-liquid ratio (S/L) of 0.5:10 m/v was maintained, with the treatment conducted at 60 °C for 1 h, followed by the addition of 1 mL of water and subsequent washing steps. The sample was called Gel_blank.

Table 2 summarizes the list of the samples obtained from the mild treatment of tanned leather with different DESs, using the sample code Gel_*x*_*y*, where *x* = 1 (DES1), 2 (DES2), and 3 (DES3) and *y* = HCl or NaOH. More in detail, the Gel_1 series gathers the samples prepared using DES1, the Gel_2 series gathers the samples prepared using DES2, and the Gel_3 series gathers the samples prepared using DES3.

**Table 2:** List of Samples Obtained after the Mild Treatment of Tanned Leather with Different DESs

sample name	mild treatment
Gel_1	DES1
Gel_1_HCl	DES1 + HCl 0.1 M
Gel_1_NaOH	DES1 + NaOH 0.1 M
Gel_2	DES2
Gel_2_HCl	DES2 + HCl 0.1 M
Gel_2_NaOH	DES2 + NaOH 0.1 M
Gel_3	DES3
Gel_3_HCl	DES3 + HCl 0.1 M
Gel_3_NaOH	DES3 + NaOH 0.1 M

#### 2.2.3 Fourier Transform Infrared Spectroscopy-Attenuated Total Reflectance

Before analyzing them, samples were dried at room temperature for 48 h. Infrared spectra were recorded by using a PerkinElmer Frontiers FTIR spectrophotometer equipped with a universal attenuated total reflectance (ATR) accessory and a triglycine sulfate TGS detector. Measurements on vegetable tanned leather (powder and fragment) and gelatin derived from the mild treatment method described above were performed in ATR mode after the background acquisition. For each sample, 128 scans were collected, averaged, and processed to generate a spectrum with a nominal resolution of 4 cm^–1^. The PerkinElmer software and a written-in-house Lab-VIEW program for peak fitting were employed to run and process spectra.^50–53^ Before spectral processing, a linear baseline was subtracted by drawing a straight line through the absorbance values at 1800 and 1480 cm^–1^. Subsequently, spectra were normalized within the 1700–1600 cm^–1^ region to minimize potential artifacts at the spectral boundaries. The second derivative of the amide I band was then computed and analyzed to determine the number and positions of the Gaussian components required for the curve fitting and deconvolution procedures.

The amide I region was specifically selected for secondary structure analysis due to its minimal interference from amino acid side chain vibrations^54^ and its greater absorbance intensity compared to the amide II (∼1540 cm^–1^) and amide III (1350–1190 cm^–1^) regions. Based on established assignments of secondary structure components within the amide I band and under the assumption of equal extinction coefficients for all structural elements, the relative proportions of secondary structures were quantified. This was achieved by calculating the ratio of the amplitude of each band, corresponding to a given secondary structure, to the sum of the amplitude of all deconvoluted amide I components.^50^

#### 2.2.4 Thermogravimetric Measurements (TG and TG/FTIR)

Before analysis, the samples were dried at room temperature for 48 h. TGA experiments were carried out on 2–5 mg of solid residue using a TA Instruments Thermobalance model Q5000IR at a rate of 10 °C/min, from room temperature to 900 °C under nitrogen flow (25 mL min^–1^). Data processing was carried out using instrument software (TA Universal Analysis). The TG analyses were performed in triplicate. In addition to standard TGA, thermogravimetric analysis coupled with Fourier transform infrared spectroscopy (TG-FTIR) was performed using a TA Instruments Q5000IR thermobalance connected to an FTIR spectrometer (Agilent Technologies). Measurements were carried out under nitrogen atmosphere (flow rate: 70 mL min^–1^) from 25 to 900 °C, with a constant heating rate of 20 °C min^–1^. FTIR spectra were collected over the range of 700–4000 cm^–1^, using a spectral resolution of 4 cm^–1^. For each analysis, approximately 8–10 mg of sample was used. To minimize interference from ambient water vapor and carbon dioxide, the FTIR optical path was continuously purged with nitrogen. A background spectrum was recorded before each run to eliminate environmental contributions and ensure the accurate detection of the evolved gases.

#### 2.2.5 Evolved Gas Analysis – Mass Spectrometry (EGA-MS)

Analyses were performed with an EGA-PY/3030D microfurnace pyrolizer (Frontier Laboratories, Japan) coupled to a 6890 Gas Chromatograph and a 5977 Mass Selective Detector single quadrupole mass spectrometer (Agilent Technologies, Palo Alto, USA). The sample (∼40 μg) was weighed into a stainless steel cup and introduced into the furnace, and the temperature was raised from 50 to 800 °C at 10 °C/min. The interface temperature was kept 100 °C above the furnace temperature up to a maximum of 280 °C. Evolved gases were carried by a helium flow (1 mL/min) to the GC injector (280 °C, split 50:1) and through a deactivated stainless-steel capillary tube (UADTM-2.5N, 3 m × 0.32 mm, Frontier Laboratories) inside the GC oven kept at 300 °C, reaching the MS detector (EI positive mode, *m*/*z* range 50–600, ion source 230 °C, quadrupole 150 °C). Thermograms were acquired in full scan mode by using MassHunter Workstation software (version 10.0, Agilent Technologies).

#### 2.2.6 Pyrolysis–Gas Chromatography–Mass Spectrometry (Py-GC-MS)

The Py-GC-MS instrumentation consisted of a Multi-Shot Pyrolizer EGA/Py-3030D microfurnace (Frontier Laboratories Ltd. Fukushima, JP) coupled to a 6890 Gas Chromatograph and a 5977 Mass Selective Detector single quadrupole mass spectrometer (Agilent Technologies, Palo Alto, USA). The GC was equipped with a split/splitless injector, a pre column (2 m, deactivated silica, i.d. 0.32 mm) and a capillary column HP-5MS Ultra Inert (stationary phase 5% diphenyl-95% dimethyl-polysiloxane, 30 m × 0.25 mm i.d., 0.25 μm, Agilent J&W, USA). The sample (∼150 μg) was weighed into a stainless steel cup and introduced into the furnace, which was maintained at a temperature of 550 °C. The pyrolysis products were eluted with a helium flow (purity 99.9995%) of 1.0 mL min^–1^ and a split ratio of 1:10. The temperature program was as follows: isothermal at 50 °C for 5 min, 10 °C min^–1^ to 310 °C, and isothermal for 30 min. The GC interface and transfer line were maintained at 280 °C throughout the analysis. The mass spectrometer operated via electron ionization (EI) at 70 eV and was equipped with a quadrupole operating in positive mode within the mass range of 35–550 *m*/*z*. The ion source temperature was set to 230 °C and the quadrupole temperature to 150 °C. Chromatograms were acquired in full scan mode using MassHunter Workstation software (version 10.0, Agilent Technologies). Peak identification was performed by comparing the mass spectra of the detected peaks with those in the NIST (National Institute of Standards and Technology, US) spectral libraries.

#### 2.2.7 Proteomics

Enzymatic digestion of samples was performed with S-Trap columns according to the protocol reported in ref 55 with slight modifications. 0.5–1 mg of sample was suspended in 25 μL of lysis buffer (10% SDS, 100 mM ammonium bicarbonate (AMBIC) and 25 μL of Milli-Q water (H_2_O)). Samples were incubated for 16 h at room temperature and then for 30 min in the ultrasonic bath. Eight μL of phosphoric acid (12%) was added to the mixture. The samples were then centrifuged at 6,000 rpm for 3 min to recover the supernatant. The pellets were discarded, and 348 μL of S-Trap buffer (100 mM AMBIC in 90% methanol/10% H_2_O) was added to load the sample onto the S-Trap membrane. The solutions were centrifuged at 6,000 rpm for 3 min to promote protein adsorption onto the membrane. Three washes with 350 μL of S-Trap buffer were performed to clean up the protein sample, followed by centrifugation at 6,000 rpm for 3 min. For enzymatic digestion, 125 μL of trypsin (0.125 μg/μL) dissolved in 50 mM AMBIC was added to each sample. The digestion reaction was performed at 37 °C overnight in a thermostatic bath. To recover the peptides after enzymatic hydrolysis, the resin was washed sequentially with 80 μL of 10 mM AMBIC in 0.2% formic acid (HCOOH), 80 μL of 0.2% HCOOH, and 80 μL of an 50% acetonitrile (ACN)/50% H_2_O/0.2% HCOOH solution. Each washing step was followed by centrifugation at 6,000 rpm for 3 min. The eluate was dried using a SpeedVac concentrator and stored at −20 °C. Peptides were desalted and concentrated on in-house C18 extraction stage tips as described by (Cappellini et al., 2012). Peptides were eluted with 10 μL of 50% ACN/0.1% HCOOH, and analyzed by LC-MS/MS on a Dionex Ultimate 3000RSLCnano system coupled to an Orbitrap Eclipse mass spectrometer (Thermo Fisher Scientific). Data were acquired by Xcalibur software (Thermo Fisher Scientific), and the raw data were processed by MaxQuant (MQ) software version 2.7.3.0 for protein identification.^56,57^ Standard parameters in the MQ searches were trypsin as the enzyme (semitrypsin when searching for backbone cleavages); 3, as the allowed number of missed cleavages; 10 ppm MS tolerance and 0.6 Da MS/MS tolerance; and peptide charge from 2+ to 3+. Minimum peptide length was set to 7; and no fixed modification was set, while oxidation of methionine, deamidation at asparagine and glutamine, hydroxylation of proline, and hydroxylation of lysine were set as variable modifications, with up to a maximum of 5 modifications per peptide. Protein identifications were supported by a false discovery rate (FDR) of 0.01 applied. Contaminant proteins were assessed using the cRAP database, which includes common laboratory contaminants. These protein hits were excluded from further analysis. To reduce the search space, searches were carried out using the UniProt reference *Bos taurus* proteome database (6,052 sequences). The extent of chemical modifications in samples was semiquantitatively assessed by a spectral counting approach,^58^ where the number of PSMs containing a specific modification on a given amino acid, as provided in the outputs of the search engine, is normalized with respect to the total number of PSMs containing that amino acid. This semiquantitative value represents the degree of modification in a sample and provides an adequate comparison between samples.^58^ Similarly, the extent of backbone cleavage in samples was evaluated as a percentage of semitryptic peptides over the total number of identified peptides, on the basis of the PSMs. The extent of modification as well as the extent of backbone cleavage in samples were calculated using home-developed command-line scripts available at https://github.com/brunellacip-prot/proteomics-mod-analyzer_public and https://github.com/brunellacip-prot/backbone_cleav_analyzer_public. The proteomics results shown were obtained by analyzing two replicates for each sample. Error bars in the graphs represent the standard error of the mean value.

#### 2.2.8 UV–vis Measurements

UV–vis measurements were performed on DESs used for multiple extraction cycles by using a Jasco V550 spectrophotometer. The spectra were acquired in the range 200–900 nm, at a speed rate of 400 nm/min. The samples, diluted 1:300 in water, were measured in a quartz cell of 1 cm length. The baseline (acquired using an empty quartz cell) was acquired under the same conditions and subtracted to each measurement.

#### 2.2.9 Adhesion tests

For the adhesion tests, gelatin was used as the adhesive immediately after the treatment (Gel_*x*, Gel_*x*_HCl, and Gel_*x*_NaOH), without undergoing any drying process. Two commercial glue samples commonly employed in restoration were used as reference standards for a comparison of the adhesive properties of the DES-treated samples: HP3, classified as rabbit glue, and BM5, classified as strong glue. Further details on HP3 and BM5 are reported in ref 59. To evaluate bonding performance, two approaches were employed: an indirect method (qualitative adhesion) and a direct method (bonding strength). In the qualitative adhesion tests, gelatin was applied (∼100 mg) as a thin layer on the surfaces of paper, glass, and wood. A second surface of paper or wood was placed on top, gently pressed by hand, and left to dry at room temperature for 30 min. After this, the two surfaces were manually separated to assess whether gelatin functioned effectively as an adhesive. Wet resistance was assessed by bonding two surfaces (paper strips, paper-to-glass, or wooden pieces) following the same procedure as that described for qualitative adhesion. The bonded specimens were then immersed in water at 25 °C, and at predetermined time intervals, detachment was monitored to evaluate the adhesive strength under wet conditions. Adhesive strength was measured using a stress-controlled Thermo Scientific HAAKE Rheostress 6000 rheometer equipped with a Peltier temperature control unit. The geometry plate–plate with 35 mm diameter uses a steel-made upper plate and a wood-made lower plate. The procedure used followed the principles of a tack test.^60^ More in detail, the upper plate was lowered until a gap of 1 mm, then, after 5 s of contact, normal strength of the upper plate was evaluated while increasing the gap at the constant speed rate of 0.1 mm/s. The measurement was conducted at 25 °C. RheoWin DataManager software was employed for data treatment.

## 3 Results and Discussion

### 3.1 Characterization of Vegetable-Tanned Leather

Before performing the treatment with DES, the leather was grated to increase the surface area and enhance the interaction with the solvent system (Figure 1SB). The study of the collagen structure after grating was evaluated by FTIR-ATR and thermogravimetric measurements (Figure 2).

**Figure 2:**
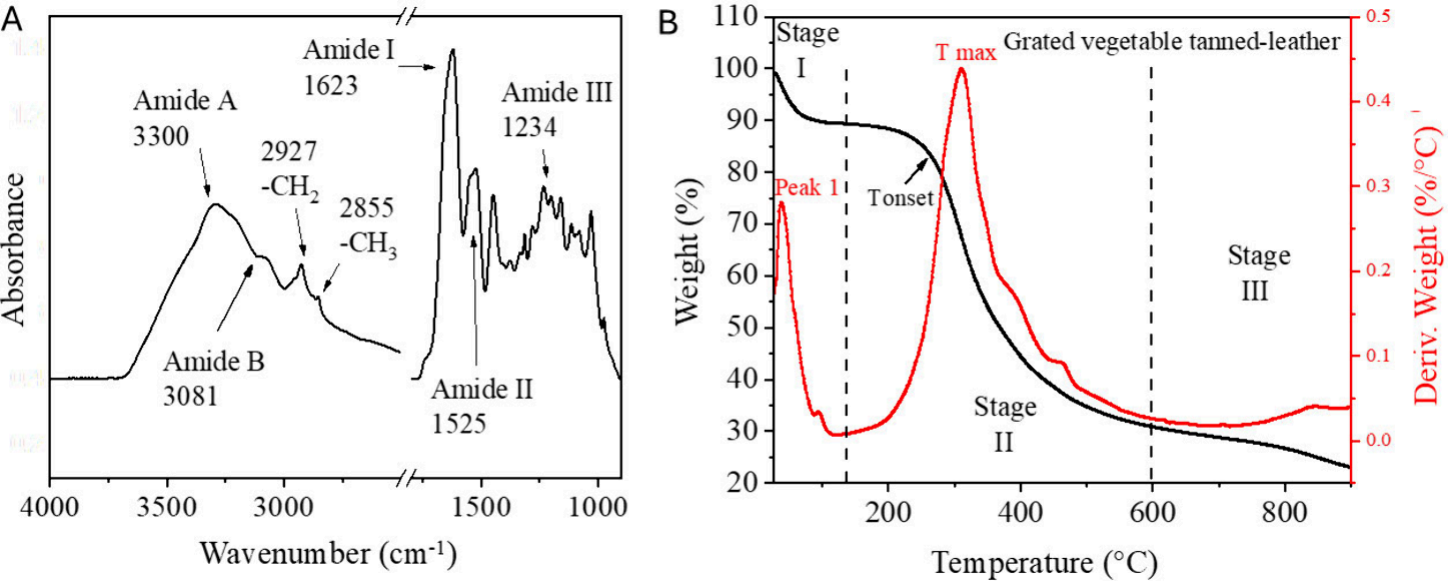
(A) ATR-FTIR spectra of the grated vegetable-tanned leather and (B) thermogravimetric curve (TG, left axis) and its derivative (DTG, right axis – red line) of the grated tanned leather under nitrogen flow at a heating rate of 10 °C/min. Peak 1 and T max are reported. The sample was analyzed in triplicate.

FTIR-ATR spectra of the leather before and after the grating process were similar to those of commercial collagen and exhibited the characteristic peaks reported in the literature (Figure 2A and Figure S2).^61^ In particular, the spectra show the typical protein absorption bands (amide A, amide B, amide I, amide II, and amide III): the broad peak at ∼3300 cm^–1^ and the shoulder at ∼3081 cm^–1^ correspond to the amide A and amide B bands, respectively, which are attributed to N–H stretching vibrations;^34,62,63^ the peaks at 2927 and 2855 cm^–1^ represent the asymmetric stretching vibrations of −CH_2_ and −CH_3_ groups, respectively;^64^ the amide I band, located at 1623 cm^–1^, is associated with the vibration of peptide C=O groups;^64^ the amide II band at 1525 cm^–1^ corresponds to the N–H bending vibration coupled with C–N stretching;^65^ the amide III band, centered at 1234 cm^–1^, is assigned to C–N stretching and N–H bending vibrations from amide linkages.^66^ To better investigate the potential changes in the protein’s structure due to the leather grating, the amide I region of the FT-IR spectra of the vegetable-tanned leather, examined both as a powder and as a fragment, was analyzed using a peak fitting procedure. The deconvolution analysis of the Amide I band for the untreated leather sample evidenced three principal contributions, with peaks at 1620 cm^–1^ and 1689 cm^–1^ assigned to β-structures, and a peak at 1660 cm^–1^ characteristic of α-helical motifs.^67,68^ In the grated leather, the same components were observed but with a slight decrease of the relative content of helix (Table 3), suggesting that the grating process induces minor alterations to the protein structure.

**Table 3:** Wavenumber, Half Height Bandwidth (HHBW), and Secondary Structure Percentages of the Amide I Component of the Samples^a^

Sample	Wavenumber (cm^–1^) (HHBW)/secondary structure percentage	
	Random and helices^a^	β structures (β-sheets/β-sheet (ap)/β-turn)
Leather as-is	1660 (40) (helix)/30%	1610 (33)/3%
		1620 (17); 1689 (18)/63%
		1672 (29)/7%
Grated leather	1662 (30) (helix)/21%	/
		1618 (32); 1693 (18)/58%
		1671 (19)/21%
Gel_blank	1652 (48)/65%	1634 (17)/8%
		1623 (23); 1694 (22)/27%
		/
Gel_1	1651 (58)/69%	1619 (32)/31%
		/
Gel_1_HCl	1659 (41)/55%	1610 (15)/32%
		1622 (27)/13%
		/
Gel_1_NaOH	1655 (49)/36%	1611 (25)/58%
		1622 (20)/6%
		/
Gel_2	1659 (33)/41%	1610 (12)/8%
		1622 (34); 1689 (26)/51%
		/
Gel_2_HCl	1660 (49)/39%	1610 (12)/8%
		1622 (34); 1689 (26)/53%
		/
Gel_2_NaOH	1661 (53)/8%	/
		1625 (40); 1689 (30)/84%
		1672 (22)/8%

a The variation coefficient for secondary structure percentage was <7% for a content of secondary structures >30% and 7–15% for a content of secondary structures <30%.^78^.

b Where not specified, this band includes random and helix structures because the peaks assigned to these two components in collagen are too close (1650–1660 cm^–1^ and 1640–1660 cm^–1^), and they may not be separated by the peak deconvolution procedure. An unambiguous assignment to the helix was considered with a narrow HHBW.

This observation was further supported by thermogravimetric analysis (TGA). Figure 2B and Table S1 show the thermogram and decomposition of grated leather. The process can be divided into three stages: stage I, ranging from 30–180 °C; stage II, from 180–600 °C; and the final stage III, from 600–900 °C. The mass loss observed in stage I is attributed to the loss of H_2_O and other volatile compounds. The actual protein thermal degradation occurs in Stage II. The onset degradation temperature (T_onset, reported in Table S1) was used to compare the thermal stability of the samples. It can be observed that the T_onset of the piece of tanned leather is lower than that of collagen,^69^ which may indicate that the vegetable tanning process has slightly weakened the collagen structure. T_onset of grated leather shows a further slight reduction, but both the leather piece and the grated leather samples exhibit a maximum decomposition rate at approximately 310 °C. Interestingly, this temperature is lower than that observed for collagen (326.1 °C), in agreement with previously published studies.^69^ Finally, the material undergoes slow decomposition until the end of the experiment. This phenomenon is likely due to processes such as the restructuring of the char, the desorption of volatile compounds previously retained within the char matrix, and similar mechanisms.^70,71^ As reported in the literature, the residue of the leather fragment and grated is around 11% higher than the collagen residue, due to the pyrolytic residue of tanning agents and collagen reticulation.^69^

### 3.2 Mild Treatment of Vegetable-Tanned Leather with DESs

The systems prepared (reported in Table 1) are homogeneous liquids exhibiting medium to low viscosity (around 0.5 Pa·s) and have been previously characterized and classified as Deep Eutectic Solvents in the literature.^18,72–75^ They were used to treat the grated vegetable-tanned leather under mild experimental conditions (*T*_1_ = 60 °C, *t*_1_ = 1 h), as reported in Figure 1.

The experimental conditions were optimized following the preliminary tests. Briefly, short treatment times and low temperatures (i.e., 30 min, 25 °C) did not sufficiently induce adhesive properties in the material. Conversely, longer times and higher temperatures (24 h, 60 °C) caused the DESs to interact strongly with collagen, leading to complete solubilization. However, the collagen recovered after dialysis did not exhibit adhesive properties under these conditions either.

The choice of experimental conditions reported in the manuscript therefore were identified as the optimal compromise to effectively promote adhesiveness, while minimizing both the amount of collagen solubilized in the DES (which would reduce the yield of the final adhesive material obtained) and the total energy consumption of the process. The energy required for the treatment (a standard hot plate requires around 0.6 kW/h to maintain a temperature of 60 °C for 1 h, with an average cost of 0.15€) is 4–6 times less than that required for the preparation of commercial adhesives from animal collagen sources (which requires 4–6 cycles of boiling the tissues in water^59^).

The supernatants isolated after the treatment appeared colored (Figure 1), indicating the presence of tannins removed from the tanned leather. The complete removal of tannins by the DES could be a promising and sustainable strategy for recovering tannins from leather processing waste and will be the focus of future investigations.

After extraction, water was added to the DES-tanned leather residue to reduce the viscosity of the system, thereby enabling centrifugation and gelatin isolation. This outcome suggests that the DES not only diminishes collagen–tannin interactions but also likely weakens collagen–collagen interactions, ultimately promoting fiber swelling.^30^

To better understand how DESs may interact with tanned leather, we refer to the existing literature on the interaction between DESs and native collagen. Both the native collagen and the DES chosen in this work contain a high density of polar functional groups (−COOH, −NH_2_, −OH).^4^ It is reasonable to hypothesize that DESs can penetrate the leather fiber network, leading to fiber swelling.^32^ To the best of our knowledge, although several studies have reported the use of LA:U (DES1) systems with native collagen,^31,34^ no specific hypotheses have been proposed regarding the interaction mechanisms between DES1 and the protein. It is well established that urea acts as a protein denaturant through hydrogen bond disruption.^76^ Likewise, lactic acid, due to its hydroxyl and carbonyl groups, may also compete with protein functional groups for hydrogen bonding.^35^ These considerations support the hypothesis that DES1 could alter the protein structure through combined polar and hydrogen interactions, which may contribute to the observed effects of our treatment.

According to the literature, the interaction between DES2 and collagen is primarily governed by the disruption of inter- and intramolecular hydrogen bonds, as well as ionic interactions among collagen chains.^32,35^ The high abundance of hydrogen and oxygen atoms in the functional groups of various amino acid residues in collagen, such as hydroxyl (−OH), carbonyl (C=O), and amine (−NH_2_) groups, provides numerous sites for hydrogen bonding with DES2 constituents. Chloride ions (from ChCl) and functional groups, such as hydroxyl and carbonyl (from LA) play a key role in the formation of these interactions. Similar interactions can be assumed for collagen in tanned leather.

The interaction between DES3 and collagen is also governed by the disruption of inter- and intramolecular hydrogen bonds within collagen fibers, followed by the formation of new hydrogen bonds between collagen and DES3.^33^ In our case, the gelatin obtained from treatment with DES3 proved extremely difficult to isolate, as it remained dissolved in the DES3 medium. This observation suggests a strong interaction between DES3 and collagen. Moreover, the small amount of gelatin that could be isolated as a residue did not exhibit adhesive properties, making it unsuitable for our intended applications. These limitations led us to refrain from further investigating the use of DES3 for the mild treatment of tanned leather.

The possibility of reusing DESs for subsequent extractions was explored, aiming to make the process more attractive to industry, in line with the principles of circular economy. More in detail, the aqueous DES solution (recovered by combining the liquid part of DES used in mild treatment and the subsequent aqueous waste obtained from the repeated washing steps) was collected. After the excess water was removed (by simply evaporation under a fumehood overnight), the resulting DES was used to perform a second cycle of mild treatment on grated leather (as described in the Materials and Methods). The procedure was successful, allowing the obtainment of new adhesive material (Gel_1_II° and Gel_2_II°). Further cycles, however, proved to be ineffective for producing a bioadhesive material. It is important to note that in all these cycles, DES was reused in its simplest form, i.e., without any purification to recover polyphenols. While this approach is advantageous for potential industrial scale-up, it results in tannin accumulation in the reused DES, as confirmed by UV–vis measurements (Figures S4). We therefore hypothesize that after the second cycle, the DES becomes “saturated” with polyphenols, rendering it unsuitable for further bioadhesive production.

Finally, the gelatinous biomaterial was treated with either 0.1 M HCl or 0.1 M NaOH in the last procedure stage, with the aim of investigating potential effects of pH on the samples’ properties.

### 3.3 Characterization of the Biomaterial

The characterization of the gelatin samples obtained after mild treatment with DES1 and DES2 is reported below. Figure 3 shows the ATR spectra of Gel_1 and Gel_2, compared with the spectra of DES1 and DES2, respectively.

**Figure 3:**
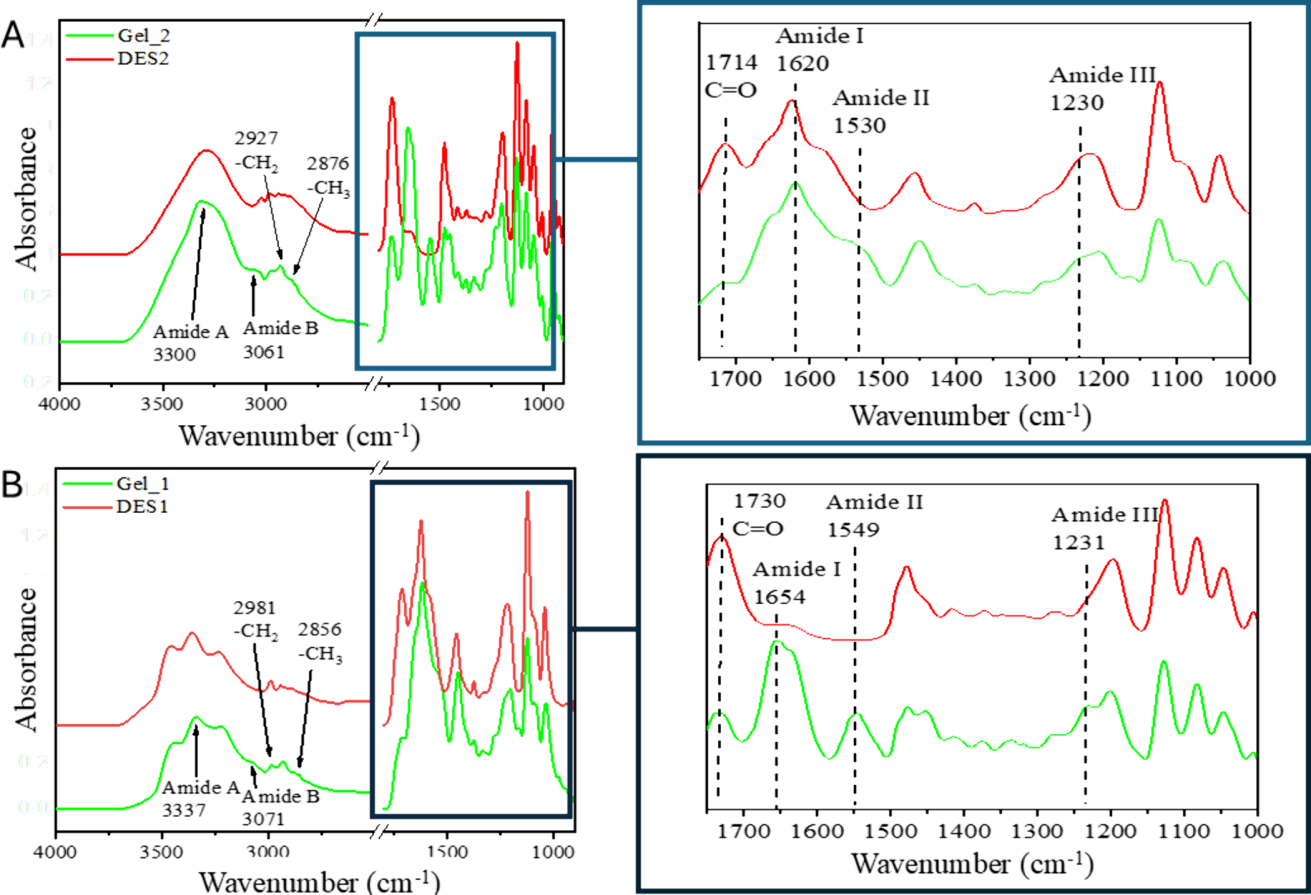
ATR-FTIR spectra of samples analyzed. (A) Dried gelatin obtained from DES1 treatment (Gel_1 - green line) compared with DES1 (red line). (B) Dried gelatin obtained from DES2 treatment (Gel_2 - green line) compared with DES2 (red line). The inlet reports the zoomed-in view of the fingerprint region. The collagen characteristics of amide bands are highlighted.

The samples Gel_1 and Gel_2 exhibit features comparable to those of grated vegetable-tanned leather, and they display the characteristic absorption bands of gelatin.^34,63,77^ Unlike the grated leather, however, the post-treatment samples exhibit an absorption peak at 1718 cm^–1^, attributable to the C=O stretching of LA present in DES1.^31^ This indicates that, despite the repeated washing in water, traces of DES1 are still present in the samples, likely due to the formation of stable bonds with collagen fibers (Figure 3A). Similarly, the samples obtained from the mild treatment using DES2 also show a peak at 1730 cm^–1^, which can be assigned to the C=O stretching of LA, confirming the presence of residual DES2 in these samples (Figure 3B).

Figure S3 reports the ATR-FTIR spectra of the samples obtained under mild treatment conditions followed by the treatment with 0.1 M HCl or 0.1 M NaOH. At first glance, the ATR-FTIR analysis does not reveal significant differences in the protein spectral features. To obtain more precise information about possible changes in the protein structure following mild treatment in neutral, acidic, and basic conditions, the amide I region of the FT-IR spectra of the dried gelatins was analyzed through peak fitting. Table 3 reports the deconvolution results of the Amide I of the spectra of the leather fragment, grated leather, Gel_blank, Gel_1, Gel_1_HCl, Gel_1_NaOH, Gel_2, Gel_2_HCl, and Gel_2_NaOH. The assignment of bands corresponding to helix and random coil structures was difficult as these bands tend to overlap due to the broad bandwidth of the random coil signal. Consequently, it was not possible to clearly distinguish between these two structures in all samples. The β-sheet assignments in Table 3 cover both parallel (p) and antiparallel (ap) β-sheets as well as β-turns. Gel_1, Gel_1_HCl, and Gel_1_NaOH show a higher content of random coil structures compared to grated leather, suggesting that treatment with DES1 induces significant structural changes in the protein, likely due to the presence of urea, which acts as a denaturant. Similarly, Gel_2 and Gel_2_HCl display an increased proportion of random structures relative to grated leather, indicating that DES2 treatment also destabilizes and alters the protein’s secondary structure. Interestingly, when NaOH was added following the treatment, the protein appears to partially regain structural order, reaching a β-structure content of 92%. Peak fitting analysis of the Amide I in the FTIR spectra shows that even the treatment without DES (Gel_blank) promotes an increase in the random protein structure. This suggests that these changes are influenced not only by the presence of DES but also by the experimental conditions used (time 60 min, temperature 60 °C).

The thermal stability of the gelatins was assessed by TGA coupled with FTIR analyses of evolved gases (Figures S5–S7).

Thermal analysis revealed that treating the grated leather with water (resulting in Gel_blank) caused only a slight thermal stabilization of the collagen degradation peak (317 °C for Gel_blank compared to 310 °C for the grated leather, as observed in the mass loss derivative). Aside from this minor shift, the thermogram shape remained essentially unchanged, indicating that water alone does not significantly affect the thermal stability of collagen. Conversely, TGA curves of the samples obtained with DES treatment present the following features: after an initial step of humidity loss (below 50 °C), all these samples present the degradation of the residue DES1 (with a maximum in DTG at around 160 °C) or DES2 (with the maximum in DTG at around 240–250 °C) respectively, followed by collagen thermal degradation.

The presence of DES was confirmed by the FTIR spectra of gases evolved at these temperatures, which showed spectral features in agreement with the nature of the products of its thermal degradation, e.g., CO_2_ (2400–2300 cm^–1^), carboxylic moieties (1800–1700 cm^–1^), NH_3_ (double peak at 965 and 930 cm^–1^ only for DES1), and C–H stretching (2800–3100 cm^–1^, only for DES2). The collagen thermal degradation was associated with the production of molecules characterized by similar spectral features, mainly CO_2_, NH_3,_ and carbonylic moieties, as well as methane (3016 and 1304 cm^–1^) and ethane (2850–3000 cm^–1^).^79^ The broad curve peak between 2600 and 3000 cm^–1^ visible above 250 °C may be due to the O–H signal of polyphenols derived from the release of vegetable tanning. The partial concomitance of DES and collagen degradation, as well as the FTIR signals overlapping and the complexity of the overall spectra, did not permit highlighting a clear onset temperature for the collagen, useful to evaluate its degree of hydrolysis after the treatment. Anyway, aware of the possible errors, we attempted to compare the collagen signals in the different samples by looking at the maximum of the DTG peak corresponding to its degradation (Table S2). Samples treated with DES1 show a slight decrease in the maximum temperature, while the degradation in the samples treated with DES2 occurs at temperatures even lower. This highlights that the treatment with the latter seems to have a higher impact on collagen thermal stability.

The thermal stabilities of the different samples were further investigated with EGA-MS, enabling molecular information to be obtained. Figure S8 reports the thermograms of Gel_1, Gel_1_HCl, and Gel_1_NaOH. In accordance with TGA-FTIR, we observed that Gel_1 still contains some residual DES, thermally decomposing in the zone below 250 °C. After 250 °C, the thermograms of Gel_1 and Gel_1_HCl are very similar, even though the maximum temperature of the main peak of Gel_1 is slightly shifted to higher temperatures, and the mass spectra present fragment ions associated with the DES. The presence of NaOH affects mass spectrometric analysis, producing a very noisy thermogram. Curves with a similar profile were obtained for Gel_2, Gel_2_HCl, and Gel_2_NaOH. To further investigate at the molecular level the characteristics of the collagen obtained with the extraction procedures described above, we thus focused on only gels washed with HCl (Gel_1_HCl and Gel_2_HCl) and not those treated with NaOH. Figure S10 shows the extracted ion thermograms (EITs) of the fragment ion *m*/*z* 154 of grated vegetable–tanned leather, Gel_blank, Gel_1_HCl, and Gel_2_HCl. Relevant pyrolysis products of proteins are diketopiperazines (DKPs), whose mass spectra are dominated by *m*/*z* 70, 111, 124, and 154.^80^ The peak of Gel_2_HCl is shifted by 10 °C to lower temperatures, meaning that the collagen in Gel_2_HCl presents a lower thermal stability, in accordance with the temperature reduction observed in the maximum peak in DTG (Table S2).

The samples were also analyzed by Py-GC-MS. The most abundant fragment ions, already identified in the EGA-MS thermograms, were selected and extracted. *m*/z 70, *m*/*z* 124, and *m*/*z* 154 are typical of DKPs,^80^ *m*/*z* 124 is also formed from the pyrolysis of catechol-derived compounds,^81^ *m*/*z* 94 is the molecular ion peak in the mass spectrum of phenol,^82^ *m*/*z* 110 is the molecular ion peak in the mass spectrum of catechol,^81^ *m*/*z* 135 is the molecular ion peak of the mass spectrum of benzothiazole, and *m*/*z* 186 is correlated to heteroaromatic compounds (see mass spectra in Figure S10). Extracted ion chromatograms are listed in Figure 4.

**Figure 4:**
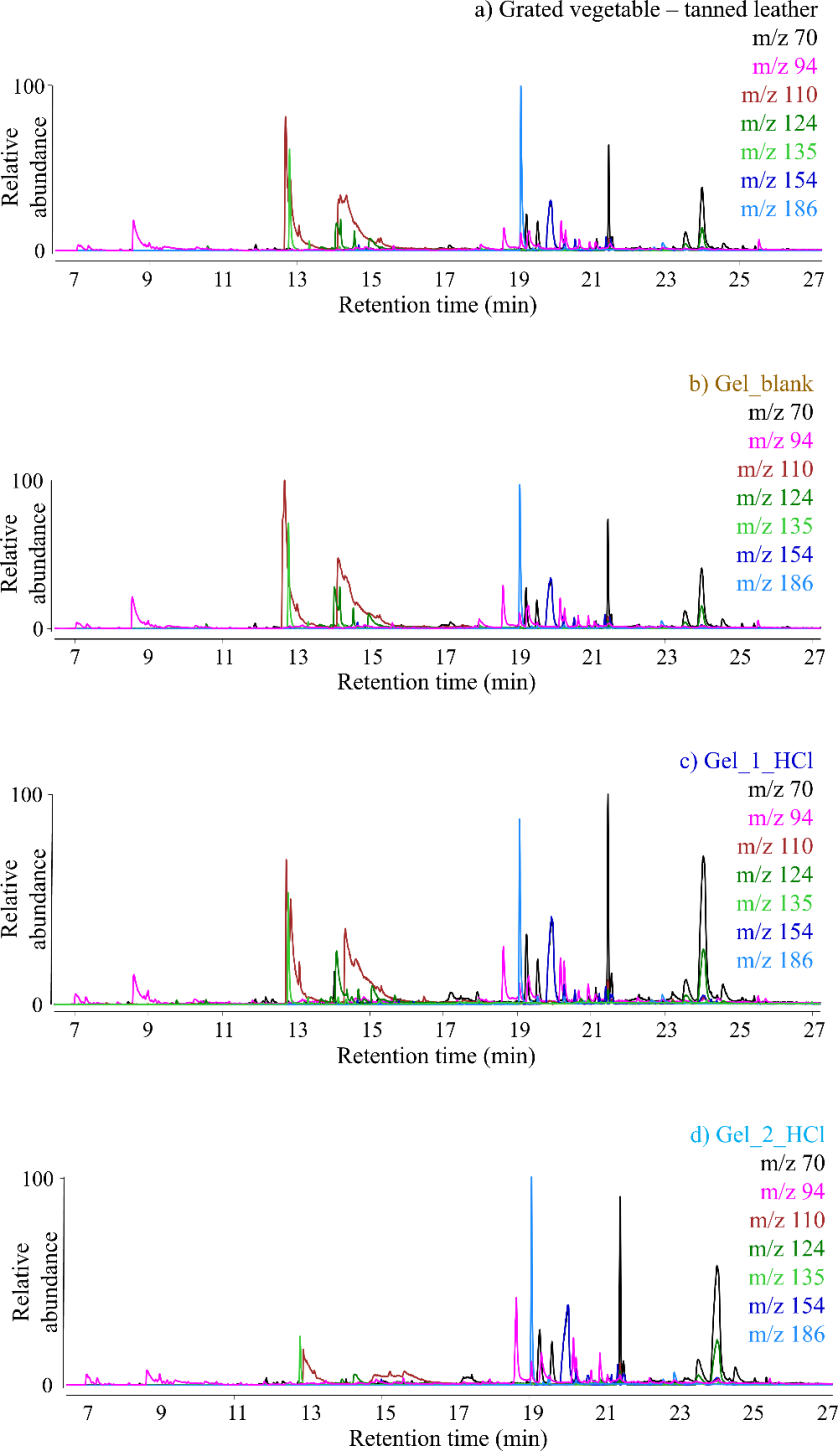
Extracted ion chromatogram (EIC) obtained with Py-GC-MS of selected ion fragments of a) grated vegetable-tanned leather, b) Gel_blank, c) Gel_1_HCl, and d) Gel_2_HCl.

EICs are divided into two regions: before and after 17 min. The first part is dominated by the peaks of phenol, catechols, benzothiazole, and catechol-derived compounds. These compounds are formed from thermal decomposition of the most thermally stable portion of collagen. All the pyrograms present several peaks whose spectra present an abundant *m*/*z* 94 fragment ion, while collagen shows only the peak of phenol with *m*/*z* 94.^80^ We hypothesize that these other peaks with *m*/*z* 94 in their mass spectral features may be associated with polyphenols derived from the leather tanning process. The second part of the EICs is dominated by the peaks of DKPs and heteroaromatic compounds: this part is characteristic of compounds formed upon thermally induced depolymerization of collagen. What stands out is that the relative intensity of the first and the second part in the EICs: in a) grated vegetable-tanned leather and b) Gel_blank the proportion is almost equal. On the contrary, the relative intensity of the second part in the EIC of Gel_1_HCl, and even more in that of Gel_2_HCl, is higher. This indicates that the collagen in the extracted gelatin is less thermally stable than that in leather, suggesting that partial hydrolysis has taken place.

In a second moment, glue samples were analyzed by a shotgun proteomics approach using LC-MS/MS after trypsin digestion and compared with the two samples of grated vegetable-tanned leather and Gel_blank, following the diagnostic strategy developed in ref 83 for the molecular characterization of commercial animal glues. Details of protein identifications are reported in Table S3.

Previous proteomics studies on collagen-based materials—including animal glues, parchments, and leathers—have shown that manufacturing processes and chemical treatments leave diagnostic molecular fingerprints that can be detected by MS-based approaches. In particular, collagen extraction and glue preparation using acidic or alkaline treatments modulate specific collagen degradation pathways, often including extensive hydrolysis of the protein backbone, resulting in lower-molecular-weight peptides. By comparing collagen samples analyzed under identical conditions but derived from different extraction and post-treatment protocols, it is possible to identify molecular signatures associated with specific degradation mechanisms and elucidate how these procedures differentially affect molecular integrity and, ultimately, adhesive performance.

Backbone cleavage or hydrolysis of the collagen polypeptide chain is a well-known feature of animal glues^83^ and reflects degradation occurring during collagen extraction and glue preparation. The backbone cleavage can be semiquantitatively evaluated since semitryptic peptides are generated upon trypsin digestion, with the trypsin cleavage site only at one end.

As shown in Figure 5a, Gel_1 and Gel_2 display comparable levels of backbone cleavage, slightly higher than those observed in the starting grated leather sample. This indicates that the DES-based extraction procedure induces some additional hydrolysis of the collagen backbone, which is in agreement with Py-GC-MS results. Notably, samples subjected to further acidic or alkaline treatments after DES extraction exhibit a markedly higher extent of backbone cleavage. Among these, the Gel_1_HCl series shows the highest values, with Gel_1_HCl reaching approximately 25%, representing an increase of about 20 percentage points relative to the starting grated leather. This pronounced effect is consistent with previous observations on animal glues, where exposure to acidic or alkaline environments during preparation has been reported to strongly promote hydrolysis of the polypeptide chain.^83^

**Figure 5:**
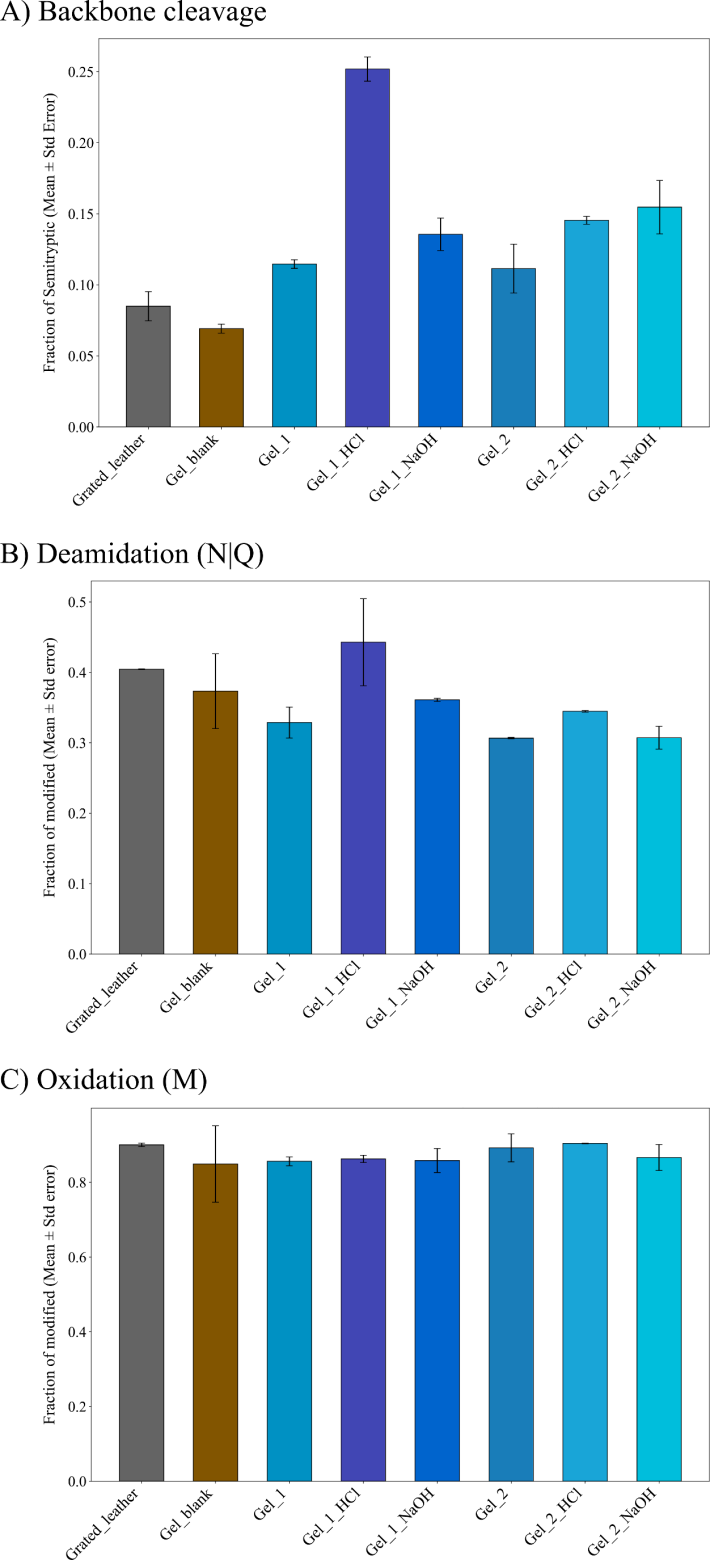
A) Extent of backbone cleavage, reported as fraction of semitryptic peptides over the total number of identified peptides (tryptic plus semitryptic). Table S4 reports the number of semitryptic peptides the backbone cleavage data are based on. B) Extent of deamidation (N|Q), reported as fraction of modified sites over detected (modified plus unmodified) ones. N|Q represents combined deamidation at both asparagine (N) and glutamine (Q) residues. C) Extent of methionine oxidation, reported as fraction of modified sites over detected (modified plus unmodified) ones. Table S5 reports the number of modified and detected amino acid sites the modification data are based on. The results shown were obtained averaging data from two replicates. Error bars in all graphs represent standard error of the mean value.

Detailed results of backbone cleavage data are reported in Table S4.

We next evaluated deamidation, a nonenzymatic modification of glutamine and asparagine residues that converts neutral amide side chains into negatively charged carboxylates, resulting in a mass shift of +0.98402 Da detectable by MS. Deamidation is particularly relevant for animal glues, as changes in charge distribution along collagen chains are known to influence physicochemical properties, like dependence of viscosity on pH.^84^

As shown in Figure 5b, Gel_1 and Gel_2 do not exhibit significant differences in the extent of deamidation. In contrast, glue samples subjected to acidic or alkaline post-treatments (Gel_*x*_HCl and Gel_*x*_NaOH) generally show slightly higher deamidation levels, approximately 5–10% greater, compared to samples treated under neutral conditions, consistent with increased hydrolysis of amide moieties at acidic and alkaline pH values.

Interestingly, the starting vegetable-tanned leather samples also display a higher extent of deamidation, approximately 10% greater than that observed in neutral gel samples. This difference may reflect preferential deamidation of collagen molecules located at the surface of the grated leather, which are more exposed to environmental factors and may be preferentially sampled during collagen solubilization prior to trypsin digestion when DES extraction is not applied.

Oxidation of methionine residues was assessed as an additional marker of collagen alteration. This modification was previously reported to be significantly abundant in commercial animal glues analyzed by Ntasi et al.^83^ In the present study, all samples, from the starting grated leather samples, exhibit very high levels of methionine oxidation, approaching 90%. This uniformly elevated level of oxidation suggests that oxidative modification was already largely present in the original vegetable-tanned leather and is therefore attributable to the tanning process rather than to the DES-based extraction or subsequent chemical treatments. Detailed results of deamidation and methionine oxidation analysis are provided in Table S5.

### 3.4 Adhesion Tests

The wet gelatin samples obtained immediately after mild treatment were tested for their adhesive properties. Adhesion was evaluated on different material combinations, including paper–paper (P–P), paper–glass (P–G), wood–wood (W–W), and wood–aluminum (W–Al) (Figures S11–S12). More specifically, paper and wood were selected as substrates, because commercial animal glues are commonly used with these materials. Since such glues are produced from leather and bone waste and our adhesive is similarly derived from vegetable-tanned leather scraps, these substrates allowed us to assess whether the DES-treated samples exhibit comparable or improved adhesion. in applications where animal glues are traditionally effective. Additionally, glass and steel were included as model substrates on which conventional animal glues usually show poor adhesion, enabling us to assess whether the DES treatment also enhances bonding on materials typically incompatible with animal-based adhesives. Steel also was tested because the upper plate used in the quantitative rheological adhesion test is made of stainless steel.

As reference materials, two glues commonly used in restoration for different applications were considered. HP3, derived from bovine hide, is typically employed for plastering operations, whereas BM5, obtained from mixed animal bones, is mainly used for carpentry applications. Generally, bone-derived glues exhibit higher initial adhesion compared to hide-derived ones, which are stiffer and more difficult to spread.^59^ A distinctive feature of animal glues, advantageous in restoration, where reversibility is required but limiting in other applications, is their solubility in hot water, which allows the adhesive to be removed. Consequently, the qualitative adhesion test was also carried out by evaluating the resistance of the DES-treated samples in hot water. Figure 6A shows the results of the adhesion test performed on the samples, testing the adhesion on dry surfaces (A) and after their immersion in hot water (60 °C) (B).

**Figure 6:**
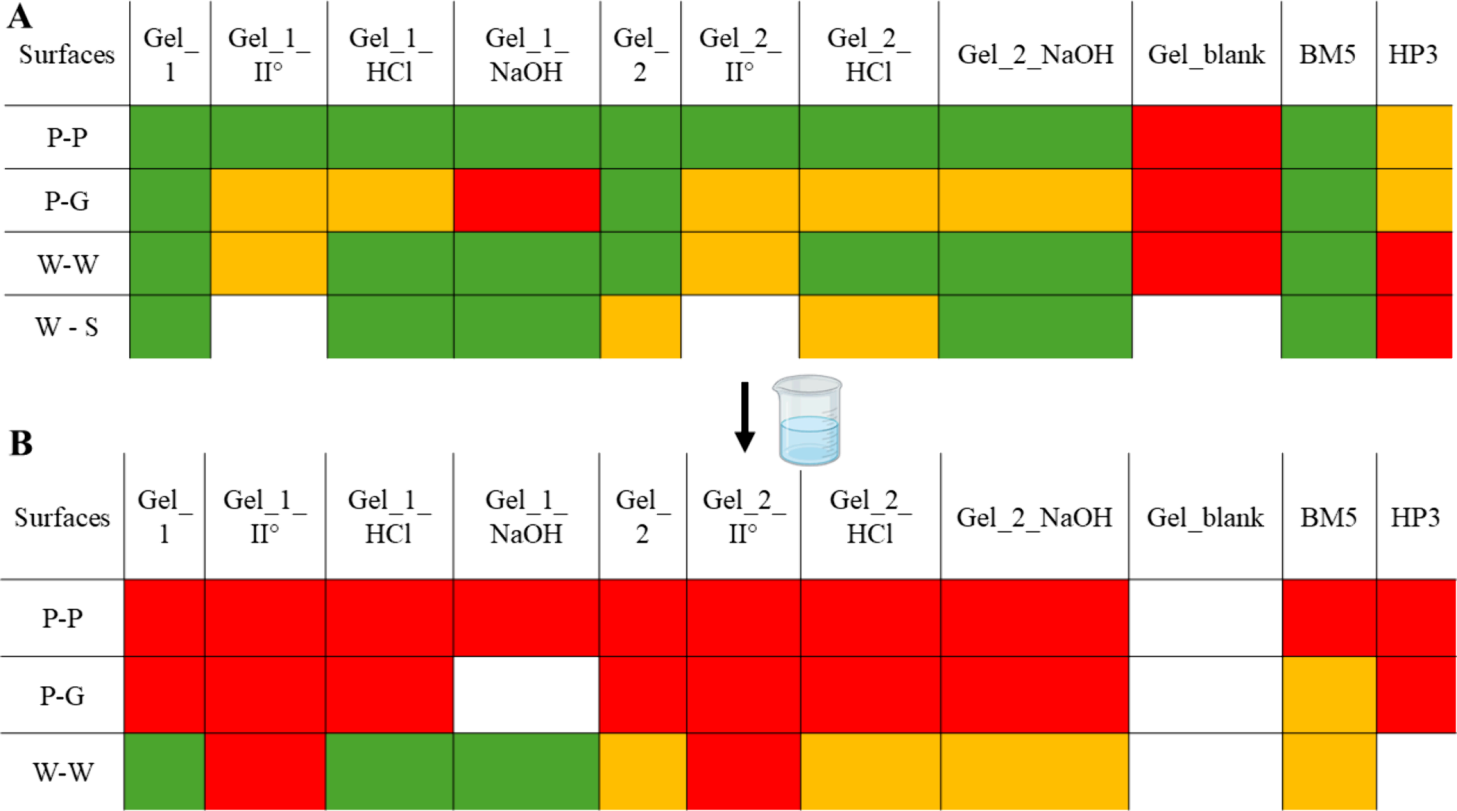
Adhesion test results using gel_1, gel_1_II°, gel_1_HCl, gel_1_NaOH, gel_2, gel_2_II°, gel_2_HCl, gel_2_NaOH, Gel_blank, and BM5 and HP3 as references. Adhesion ability between surface pairs: paper–paper (P–P), paper–glass (P–G), wood–wood (W–W), and wood-steel (W–S). A) Test made on dry surfaces. Color coding: green = strong adhesion, yellow = weak adhesion, and red = no adhesion. B) Adhesion after immersion in hot water. Color coding: green = samples that remained attached after more than 3 days in water at 60 °C; yellow = samples detached after approximately 1 min; red = samples detached immediately upon immersion.

The first important observation in our samples is that Gel_blank exhibits no adhesive properties, underscoring the essential role of DES in producing adhesive materials. The other samples, instead, show medium-to-good adhesive properties for the attachment of dry surfaces, except for Gel_1_NaOH which failed to glue paper and glass. The gels recovered after the second cycle of DES reuse (Gel_1_II° and Gel_2_II°) show good adhesive properties for gluing paper–paper surfaces and medium properties on the other surfaces, highlighting the potentialities in DES reuse for multiple cycles of treatment. The best samples (highlighted green in the table) were further tested for water resistance by immersion in hot water (Figure 6B). Notably, three samples (Gel_1, Gel_1_HCl, and Gel_1_NaOH) exhibit outstanding water resistance for gluing wood surfaces, which remained firmly adhered even after 3 days of immersion in hot water. According to the literature, collagen-based adhesives typically face significant challenges compared to their synthetic counterparts, particularly in terms of adhesion strength and water resistance.^43,47^ Numerous studies have focused on the improvement of the performance of collagen adhesives, adopting strategies as the incorporation of silane coupling agents and cross-linking technologies.^43,44,85^ In contrast to these approaches, the gelatins obtained through our DES-based mild treatment were used as adhesives without any further modification. Probably, the presence of tannins in our matrix, with their well-documented ability to enhance collagen water resistance,^41,43,47^ promotes the improvement of the overall adhesive properties of the material.

The adhesive properties of the materials for gluing wood-steel surfaces were also tested through a tack test, using a rheometer.^60^ To compare the tack properties of the investigated samples, the parameter Δ*F* (defined as the difference between F_peak and *F*_0_ values) was considered, where *F*_0_ is the initial normal force at contact and F_peak is the maximum detachment force (Table 4). This correction was necessary, because the initial *F*_0_ varied among samples. Such variations are not purely instrumental but reflect intrinsic differences in gel deformability and viscoelastic response: softer gels exhibit lower *F*_0_ because they deform more easily under contact, whereas stiffer gels resist deformation and thus show a higher *F*_0_.^86^ Since *F*_0_ directly contributes to the absolute detachment force, using uncorrected values would lead to biased conclusions. By subtraction of *F*_0_, Δ*F* isolates the additional force developed during detachment, providing a more reliable comparison across formulations and treatments. The tack test curves revealed clear differences in adhesion behavior among the samples (see Figure S12). All profiles displayed the typical viscoelastic adhesive response, with an initial increase in normal force followed by a detachment peak and decay.^87^ Nevertheless, the shape and stability of the curves strongly depended on the sample formulation and chemical environment. Looking at the results, we observed that Gel_1 exhibited a sharper, more pronounced peak and higher Δ*F* value compared to Gel_2, suggesting a stronger cohesive network and well-defined detachment profile. Acidic treatment (HCl) weakened the adhesive response, especially for Gel_2_HCl (Δ*F* ≈ 6), where the detachment peak was smoother. Gel_1_HCl, the effect was milder (Δ*F* ≈ 13), suggesting higher resistance of Gel_1 to acid-induced destabilization. This reduction in adhesion is likely due to the protonation of functional groups, which decreases electrostatic interactions and weakens the gel network. On the other hand, alkaline treatment (NaOH) produced the most distinctive profiles. In Gel_1_NaOH, detachment was accompanied by oscillations, pointing to discontinuous failure with multiple breakage; still, the corrected force was significant (Δ*F* ≈ 11). In Gel_2_NaOH, the profiles displayed sharp peaks and Δ*F* ≈ 17 values comparable to Gel_1 (Δ*F* ≈ 17). These results suggest that alkaline conditions may favor structural rearrangements, such as deprotonation and enhanced ionic interactions, which strengthen and diversify adhesive contacts.

**Table 4:**
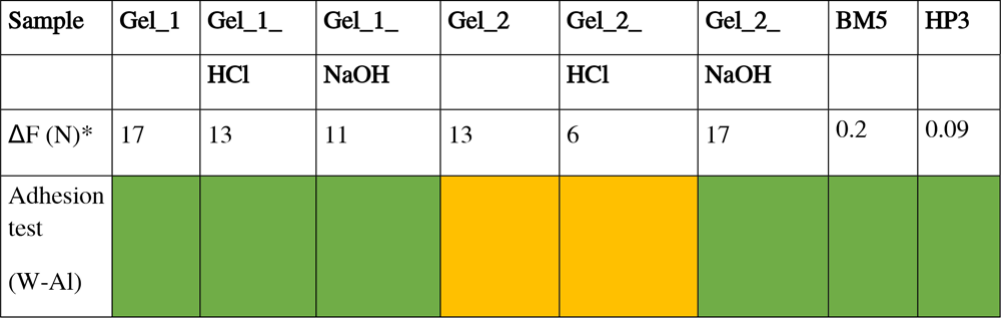
Difference between the Force at Peak Maximum and the Initial Force (Δ*F*) Obtained in the Tack Test Compared with the Results Obtained in the Adhesion Tests^a^

a The results refer to adhesive properties on wood-steel surfaces.

* Values reported with an error of ± 3 N for Gel_x series and ± 0.02 N for commercial glues.

The Δ*F*_N_ value obtained from the tack test measurements on the two commercial animal glue samples (BM5 and HP3, visible in Table 4) is significantly lower than that of the samples treated with DES. This confirms that DES treatment alters fundamental parameters governing the adhesion mechanism. Specifically, the presence of DES may induce a structural reorganization at the level of the tertiary structure, influencing protein aggregation and increasing the availability of functional groups capable of interacting with the substrate surface, thereby enhancing adhesive performance. These changes impact not only the adhesion to the substrate but also the material’s internal cohesion. The residual tannins within the protein matrix in the DES-treated samples likely strengthen cohesive forces, thanks to tannins’ ability to form multiple hydrogen bonds and π–π interactions with polypeptide chains. This results in a more robust internal network capable of dissipating more energy during debonding. Furthermore, DES may reorganize protein chains, by altering their flexibility, the distribution of functional groups, and the degree of plasticization. The combined effect of enhanced internal cohesion and a more reactive interface toward the substrate leads to a marked increase in adhesive strength. It is also worth noting that the concentration used for the commercial glues corresponds to the concentration employed in their typical applications, whereas the sample obtained through the DES treatment is likely more concentrated. This higher concentration may contribute to increase the adhesion observed. While these hypotheses could be further supported by surface tension and contact angle measurements, such analyses are currently unfeasible due to the extremely high viscosity of the samples obtained after the DES treatment, which hinders reliable testing with these techniques.

These quantitative results from the tack tests are consistent with the observations from preliminary manual detachment tests, where the adhesive behavior was assessed qualitatively. In the manual tests, it was evident that Gel_2 and Gel_2_HCl exhibited noticeably lower adhesion compared to that of the other samples. The Δ*F* measurements confirm this trend, providing a more precise and reproducible evaluation of the relative adhesive strength across all of the samples and treatments. Notably, the quantitative approach also reveals subtle differences that were not apparent in the manual tests, such as the enhanced adhesion of Gel_2_NaOH, which could not be captured by qualitative assessment alone.

Differences in adhesion performance can be discussed by looking at the issues from different angles. Thermoanalytical techniques (TGA-FTIR, EGA-MS, and Py-GC-MS) highlight the collagen hydrolysis in Gel 1_x and Gel 2_x with respect to the grated leather. From this perspective, we could say that treatment with both DESs promotes the unravelling of collagen fibers through the destruction of inter- and intramolecular bonds, followed by a certain degree of hydrolysis of the polypeptide chains. In particular, samples coming from treatment with DES2 (Gel_2_x series) are more thermally destabilized than samples treated with DES1 (Gel_1_x series). Proteomic analyses revealed substantial similarity between Gel_1 and Gel_2 samples at the level of the covalent structure. Importantly, no significant chemical modification of collagen due to DES treatment was observed, although the peak fitting analysis indicates that all samples are predominantly composed of random coil and β-structures, whereas leather prior to extraction has a less random structure. Moreover, we observed that acidic and basic treatments induce a higher degree of hydrolysis than the neutral gel; however, this difference was not reflected in the adhesive properties.

It is important to note that no direct correlation can be established between the secondary structure of the protein and the adhesive performance of the DES-treated samples. The control sample (Gel_blank), treated under identical conditions but without DES, displays a secondary structure profile comparable to that of the DES-treated samples yet shows no adhesion. This clearly indicates that secondary structure modifications alone do not govern adhesive strength, which instead likely arises from DES-mediated interactions and from the synergistic contribution of tannins, affecting cohesion and interfacial bonding. Therefore, the differences in adhesive performance observed among the samples cannot be ascribed solely to variations in secondary structure, in agreement with the findings reported by Pulidori et al.^59^

### 3.5 Process Sustainability

The sustainability of the process was evaluated using *Path2Green*, one of the most recent metrics, which gives a comprehensive method evaluation of the base of the 12 Green Chemistry principles.^88^ The pictogram obtained with the mobile app proposed by the authors is reported in Figure 7, and the scores used to build it are reported in Table S6. The pictogram displays the 12 icons relative to the 12 green principles and highlights with a colorimetric scale the process adherence to each principle: red, yellow, and green to indicate poor, neutral, or good adherence, respectively. The final process score, calculated by weighing the score of each principle for its environmental, social, and economic impacts, is reported in the center of the figure.

**Figure 7:**
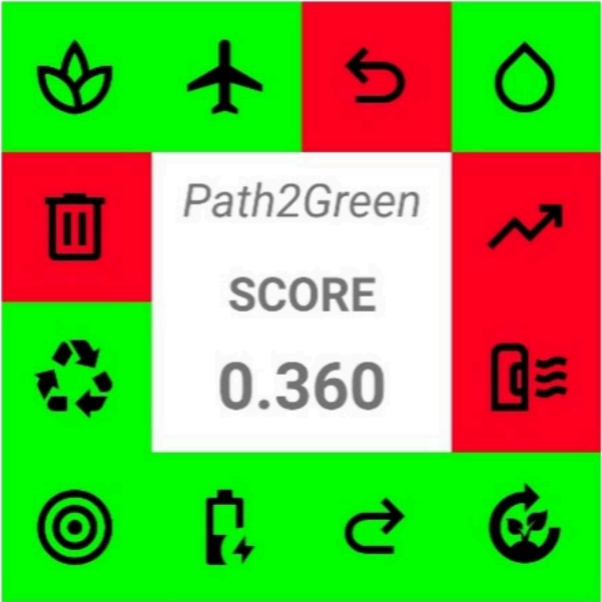
Pictogram created with the App Path2Green, highlighting the sustainability of the process.

Our process displays a final score of 0.360 on a scale going from completely negative −1 to completely positive +1. Its main strength points rely mainly on the biomass selected (Principles 1–2), the treatment conditions (Principles 4, 7, and 9), and the various application fields (Principle 10). More in detail, the leather pieces are waste biomasses from the tanning industry of the Zabri tannery, which is located in the Tuscany tanning district, one of the main areas for leather processing on a national and international level. The valorization of waste biomasses proposed in this work is therefore of great interest from several points of view, including environmental and economic sustainability (the energy required is around 4–6 times lower than the standard animal glue production) and the social impact of tanneries on the territory, as shown by the high score obtained from the evaluation of Principles 1 and 2. The solvents chosen for the treatment were DESs composed of biodegradable and economic components, and this allowed us to maximize the score for Principle 4. The process required a much lower energy consumption in comparison with other procedures necessary to produce glue from animal sources,^89,90^ with a consequent good impact on environmental and economic sustainability. After the DES-based treatment, product purification was necessary, but water was used as solvent (Principle 6), and no other post-treatment was necessary (Principle 8). The final material presented excellent qualities, providing complete valorization of the starting biomasses (Principle 7). Moreover, considering the antimicrobial properties of tannins,^49^ the biomaterial obtained has potential applications as antimicrobial glues in several fields, e.g., restoration, material science, biomedical field, or manufacturing (Principle 10). The possibility of reusing DES for consecutive treatments is reflected in the maximization of Principle 11.

The weaker points of the process are related to Principles 3, 5, 6, and 12. As a matter of fact, the biomass was subjected to physical pretreatment (which has a slightly negative impact on the process score reflected in Principle 3), even though pretreatments with biological and hazardous chemicals were carefully avoided. Besides, the procedure was performed on a scale laboratory; therefore, it was performed in batches (−1 at Principle 5), and water for purification of the material was needed (−0.5 at Principle 6). Waste management (Principle 12) is currently a negative point but with good potential for improvement. The possibility of reusing DES for multiple leather treatment cycles and ongoing studies on the tannins’ recovery from DES are the starting points for desirable waste reduction. Moreover, seeing the good premises found in this work, we believe that future optimization on a larger scale can be of interest, with a view to achieving a competitive process at an industrial level.

## 4 Conclusions

The results presented here demonstrate, for the first time, that NADES (lactic acid and urea in a molar ratio of 2:1, DES1, and choline chloride:lactic acid in a molar ratio of 1:1, DES2) can be employed to gently modify vegetable-tanned leather scraps (wastes of a local industry), leading to the production of a collagen-based biomaterial with adhesive properties. The process’s greatest strengths lie in the use of green solvents (NADES), the complete valorization of waste biomass, and the low energy required for treatment (1 h, 60 °C).

Spectroscopic (FTIR-ATR), thermoanalytic (TGA-FTIR, EGA-MS, Py-GC-MS), and proteomic techniques gave molecular insights into changes induced in collagen by the treatment. Specifically, we observed that the experimental treatment conditions caused a reduction in helices across all samples, accompanied by an increase in random-coil secondary structures. TGA-FTIR analyses revealed that a portion of DES remains tied in the final gelatin samples. Meanwhile, EGA-MS, Py-GC-MS and proteomics techniques showed that the presence of DES leads to a decrease in collagen thermal stability (higher in DES 2 with respect to DES 1), likely due to partial backbone cleavage, which, in any case, did not exceed 25%.

The DES presence was essential for the final properties of the materials, which indeed showed a good adhesive performance (especially on organic surfaces such as wood or paper). The intrinsic presence of tannins in the material (which come from the vegetable tanning process carried out on the raw leather) gave it additional antibacterial and water-resistant properties, making it an attractive alternative to common commercial glues of animal origin. Preliminary tests showed that the obtainment of adhesive materials was possible also using recycled DES.

Overall, the process described represents a first step toward a more complete recovery and reuse of waste from the tanning industry.

## Supplementary Material



## Data Availability

All the experimental data are reported in the main text or in the Supporting Information. Additional information will be made available upon request.

## Notes

Additional spectra and thermograms (FTIR spectra, TGA data, total and extracted ion thermograms, proteomics analysis), and samples photographs (PDF)
