# Supplementary material for: From Waste to Function: Valorization of Collagen-Based Wastes with Natural Deep Eutectic Solvents for Bioadhesive Applications
Source: ACS Sustain Chem Eng. 2026 Feb 3;14(6):2994–3010. doi: 10.1021/acssuschemeng.5c11526 (PMC12918149; doi:10.1021/acssuschemeng.5c11526)
Supplement: Supplementary file 1 [file sc5c11526_si_001.pdf]

SUPPLEMENTARY MATERIAL

From waste to function: valorization of collagen-based wastes with Natural Deep Eutectic Solvents for bioadhesive applications

Chiara Pelosi† [a] , Eleonora Micheli‡ [a], Elena Pulidori† [*], Giulia Caroti†, Brunella Cipolletta†‡, Beatrice Campanella‡, Iacopo Corsi‡‡, Silvia Pizzimenti†‡, Leila Birolo†‡, Ilaria Bonaduce†, Celia Duce†, Emilia Bramanti‡.

†Affiliation 1: Department of Chemistry and Industrial Chemistry, University of Pisa, Via G. Moruzzi 13, 56124 Pisa, Italy.

‡Affiliation 2: Institute of Chemistry of Organometallic Compounds, National Research Council, Via G. Moruzzi 1, 56124 Pisa, Italy.

†‡Affiliation 3: Department of Chemical Science, University of Naples Federico II, Strada Comunale Cinthia 26, 80126, Napoli, Italy.

‡‡Affiliation 4: Conceria Zabri S.p.A., Fucecchio, Italy.

[a]Chiara Pelosi and Eleonora Micheli equally contributed to the work.

Corresponding Author

*Elena Pulidori elena.pulidori@unipi.it, +39 0502219265.

TABLE OF CONTENTS

Figure S 1: Vegetable-tanned leather as supplied by the tannery (A) and after grating into small pieces (B)........ S3
Figure S 2: ATR-FTIR spectrum of the collagen (purple line), vegetable-tanned leather sample as provided by the supplier (orange line) and grated vegetable-tanned leather (black line)........ S3
Table S 1: TGA analysis of the samples: as-received tanned leather, grated tanned leather, Gel_1, Gel_1_HCl, Gel_1_NaOH, Gel_2, gel_2_HCl, and Gel_2_NaOH........S3
Figure S 3: ATR-FTIR spectra of samples analyzed........ S4
Figure S 4: UV-Vis of DES 1 (on the left) and DES 2 (on the right) used in subsequent cycles........S4
Figure S 5: TGA of the samples Gel_1, Gel_1 HCl, Gel_1_NaOH, Gel_2, Gel_2 HCl, Gel_2_NaOH........ S5
Figure S 6: FTIR spectrum of the gases evolved during thermal degradation of Gel_1 at 163°C (A) and at 323°C (B), Gel_1_HCl at 210°C (C) and at 310°C (D) and Gel_1_NaOH at 210°C (E) and at 315°C (F), under nitrogen flow at a heating rate of 20 °C/min........ S6
Figure S 7: FTIR spectrum of the gases evolved during thermal degradation of Gel_2 at 220°C (A) and at 280°C (B), Gel_2_HCl at 220°C (C), and at 280°C (D) and Gel_2_NaOH at 220°C (E) and at 280°C (F), under nitrogen flow at a heating rate of 20 °C/min........ S7
Table S 2: Maximum temperature in DTG associated with collagen degradation........S7
Figure S 8: Total Ion Thermograms (TITs) of Gel_1 (green), Gel_1_HCl (blue) and Gel_1_NaOH (pink)........ S8
Figure S 9: Extracted Ion Thermograms (EITs) of fragment ion m/z 154 of grated vegetable – tanned leather (black), Gel_blank (brown), Gel_1_HCl (blue) and Gel_2_HCl (light blue)........ S8
Figure S 10: Mass spectra of heteroaromatic compounds from EICs in Fig 4 at a) 19.1 min and b) 22.9 min........ S9
Table S 3: Summary of protein identification results........*S10*
Table S 4: Overall extent of backbone cleavage........S11
Table S 5: Analysis of protein modification extent........*S11*
Figure S11: Adhesion test with Gel_1 as bioahdesive S12
Figure S12: Adhesion test with control experiment S12
Figure S 13: Tack test made on the samples to test the adhesive strength on steel-wood surfaces........S12
Table S 6: Scores attributed to each principle to evaluate the process greenness, made according to Path2Green application S13

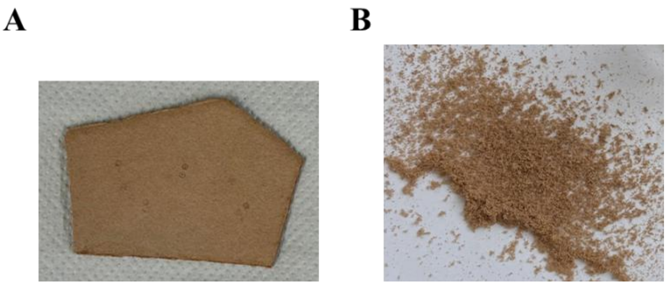

Figure S 1: Vegetable-tanned leather as supplied by the tannery (A) and after grating into small pieces (B).

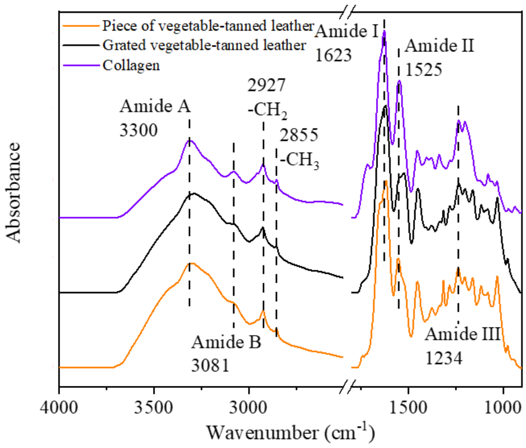

Figure S 2: ATR-FTIR spectrum of the collagen (purple line), vegetable-tanned leather sample as provided by the supplier (orange line) and grated vegetable-tanned leather (black line).
Characteristic bands associated with collagen structure are highlighted.

Table S 4: TGA analysis of the samples: collagen, as-received tanned leather and grated tanned leather.

						**Mass loss (wt%)**		
**Sample**	**T_onset (°C)**	**T (1° Peak) (°C)**	**T (2° Peak) (°C)**	**T max (°C)**	**Residue Mass % (T=900°C)**	**Stage I (25-200°C)**	**Stage II (200-600°C)**	**Stage III (600-900°C)**
Collagen	299.3	38.3	169.7	326.1	13	32	52	3
Leather as recived	268.8	46.2	/	309.6	26	15	54	5
Grated leather	263.7	39.1	/	310.3	23	11	59	7

*Error (expressed as ± standard deviation) was evaluated by performing in triplicate representative samples. More in detail, error in the temperatures was ± 0.3°C, and in the mass losses was ± 4%.

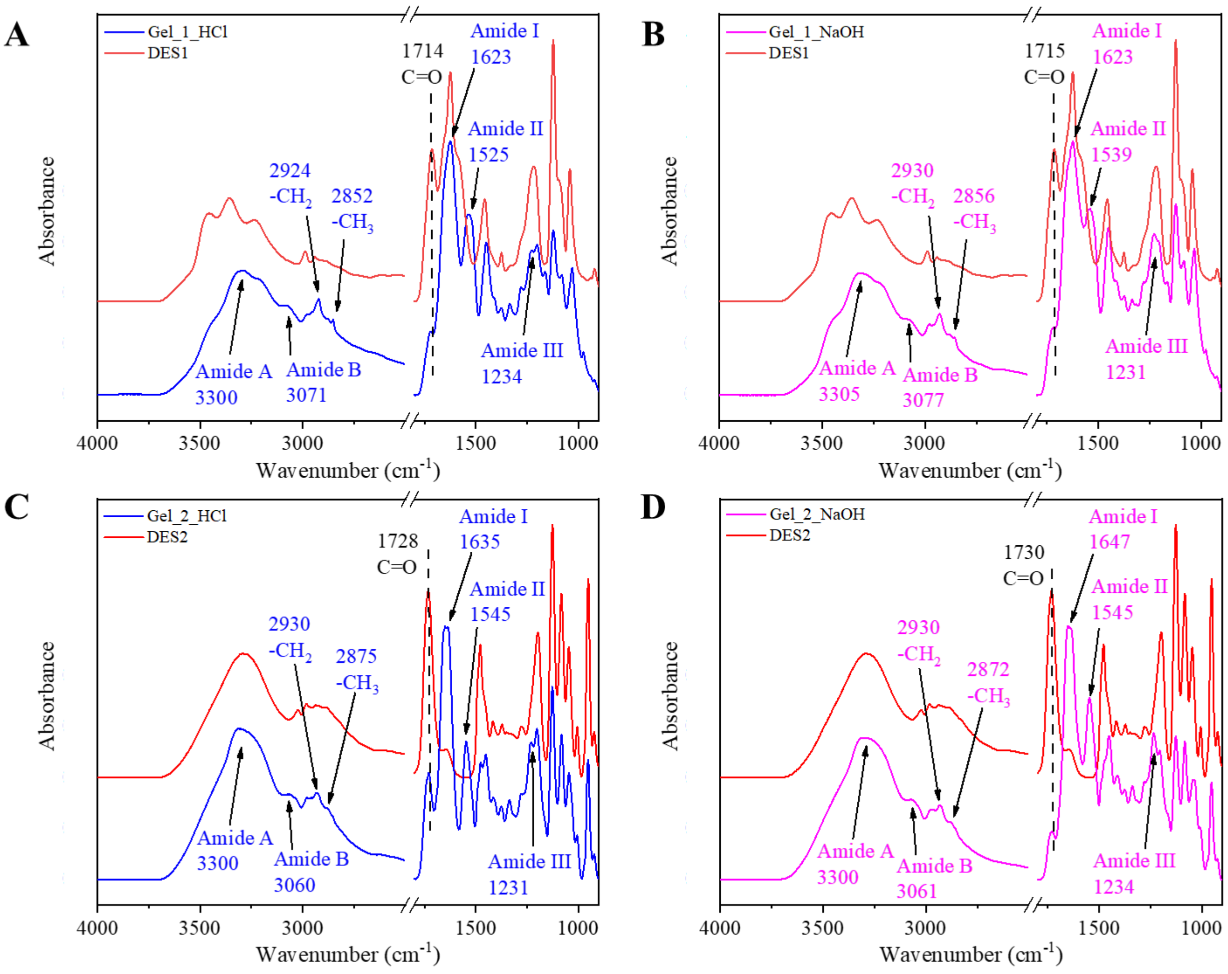

Figure S 3: ATR-FTIR spectra of samples analyzed.

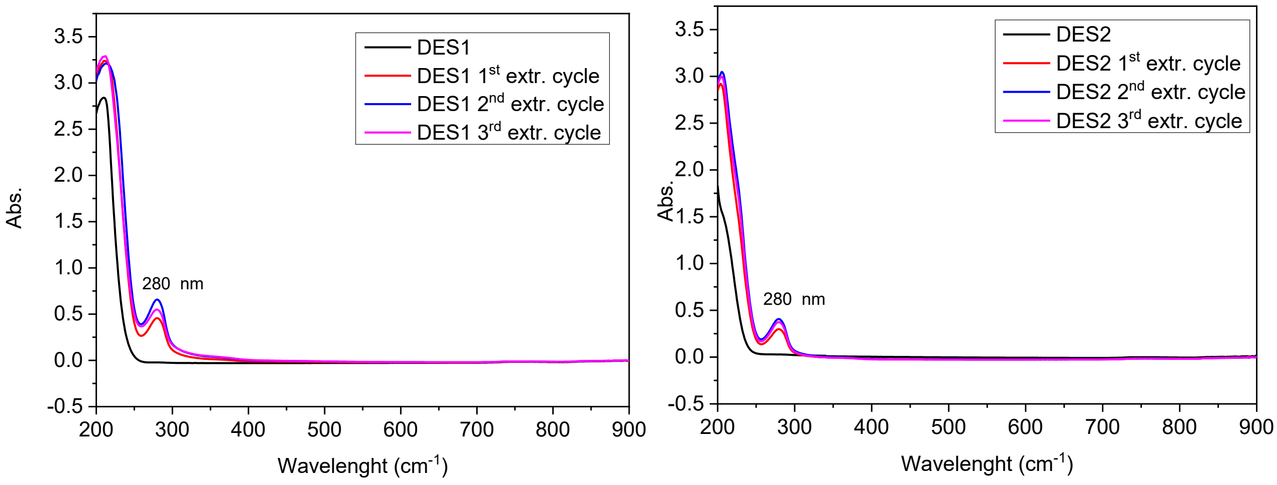

Figure S 4: UV-Vis of DES 1 (on the left) and DES 2 (on the right) used in subsequent cycles.

Samples are diluted 1:300 in water. The signal at 280 nm of mimosa and quebracho extracts containing tannins are visible (DOI: http://dx.doi.org/10.1016/j.indcrop.2016.07.022) while the signal of chestnuts extracts and the other signals of previous extracts are covered by the signal of DES.

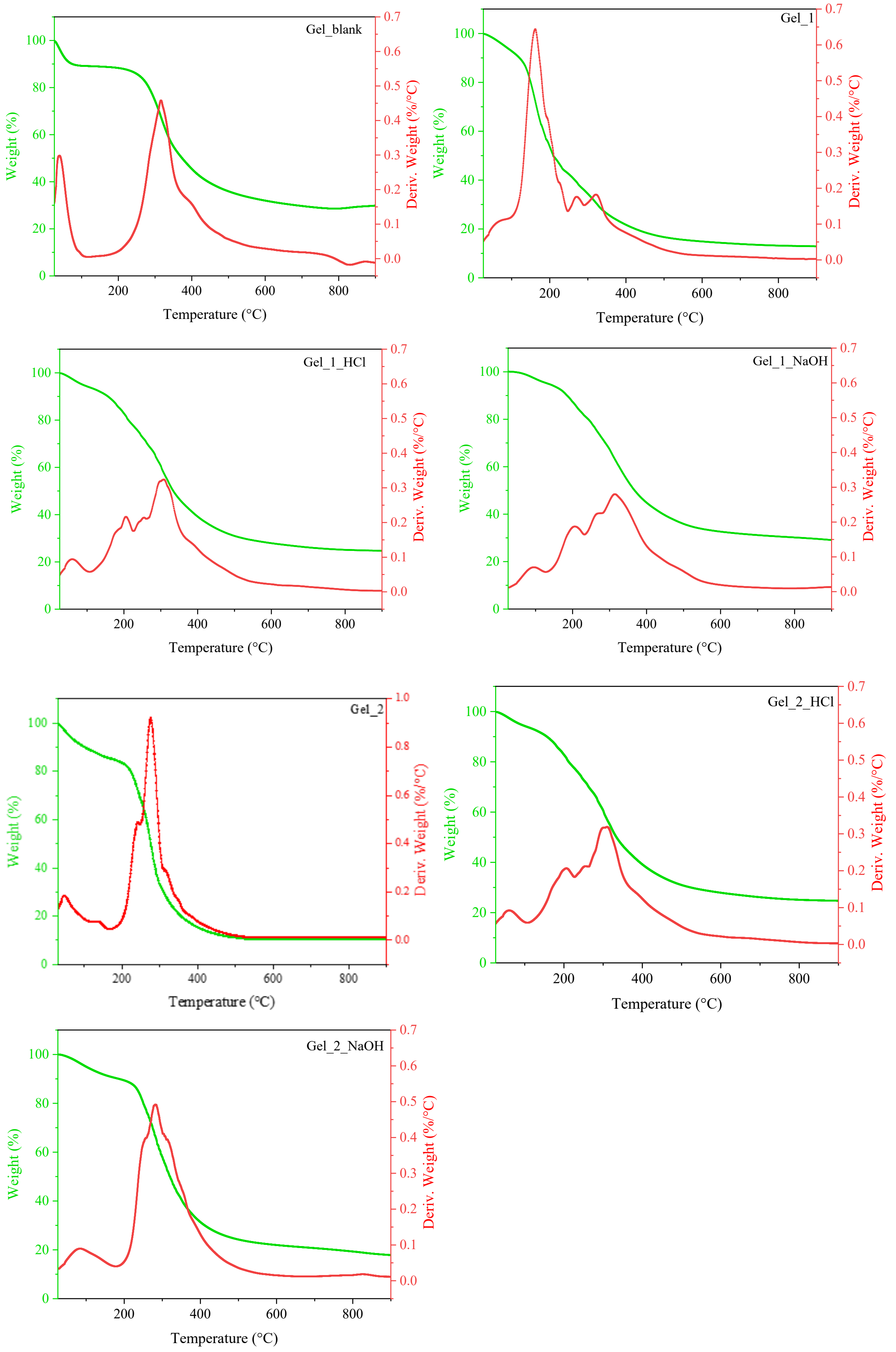

Figure S 5: TGA of the samples Gel_blank, Gel_1, Gel_1 HCl, Gel_1_NaOH, Gel_2, Gel_2 HCl, Gel_2_NaOH.

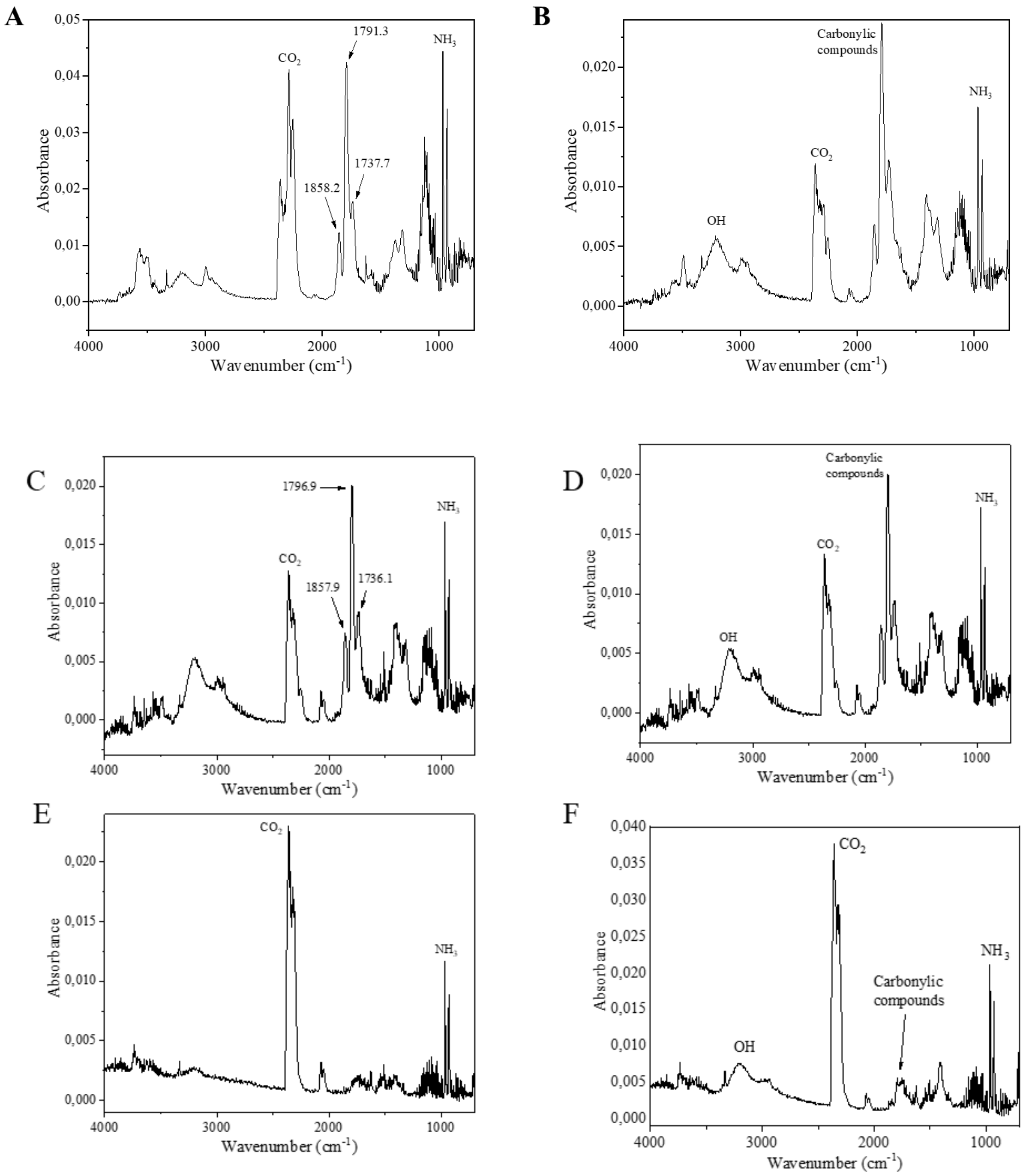

Figure S 6: FTIR spectrum of the gases evolved during thermal degradation of Gel_1 at 163°C (A) and at 323°C (B), Gel_1_HCl at 210°C (C) and at 310°C (D) and Gel_1_NaOH at 210°C (E) and at 315°C (F), under nitrogen flow at a heating rate of 20 °C/min.

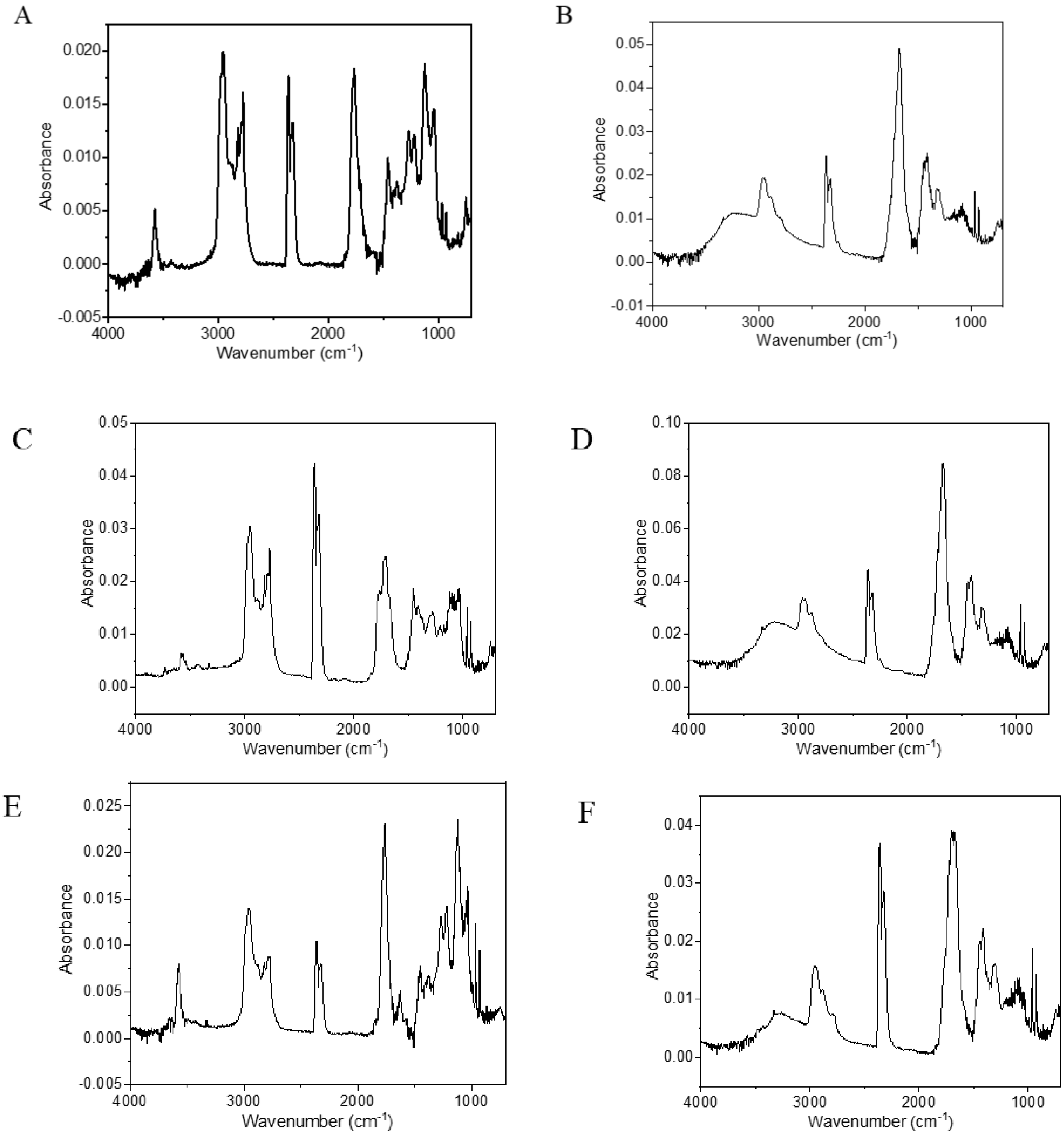

Figure S 7: FTIR spectrum of the gases evolved during thermal degradation of Gel_2 at 220°C (A) and at 280°C (B), Gel_2_HCl at 220°C (C), and at 280°C (D) and Gel_2_NaOH at 220°C (E) and at 280°C (F), under nitrogen flow at a heating rate of 20 °C/min.

Table S 5: Maximum temperature in DTG associated with collagen degradation.

**Sample**	**Collag en**	**Leather as recived**	**Grate d leathe r**	**Gel-blank**	**Gel_1**	**Gel_1_HCl**	**Gel_1_NaO H**	**Gel_2**	**Gel_2_HCl**	**Gel_2_NaOH**
T max DTG (°C)	326	310	310	317	302	301	314	275	283	282

*Error (< ±2°C, expressed as ± standard deviation) was evaluated by performing in triplicate representative samples.

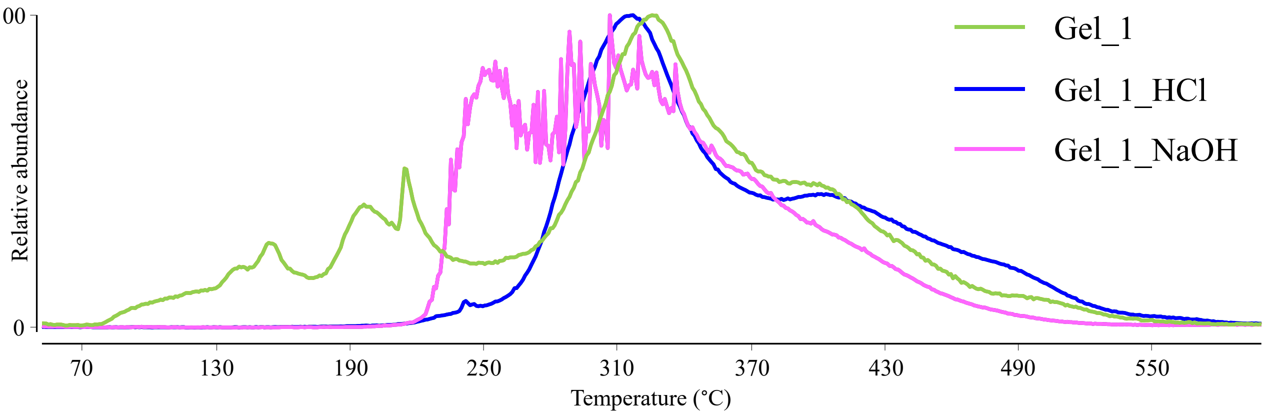

Figure S 8: Total Ion Thermograms (TITs) of Gel_1 (green), Gel_1_HCl (blue) and Gel_1_NaOH (pink).

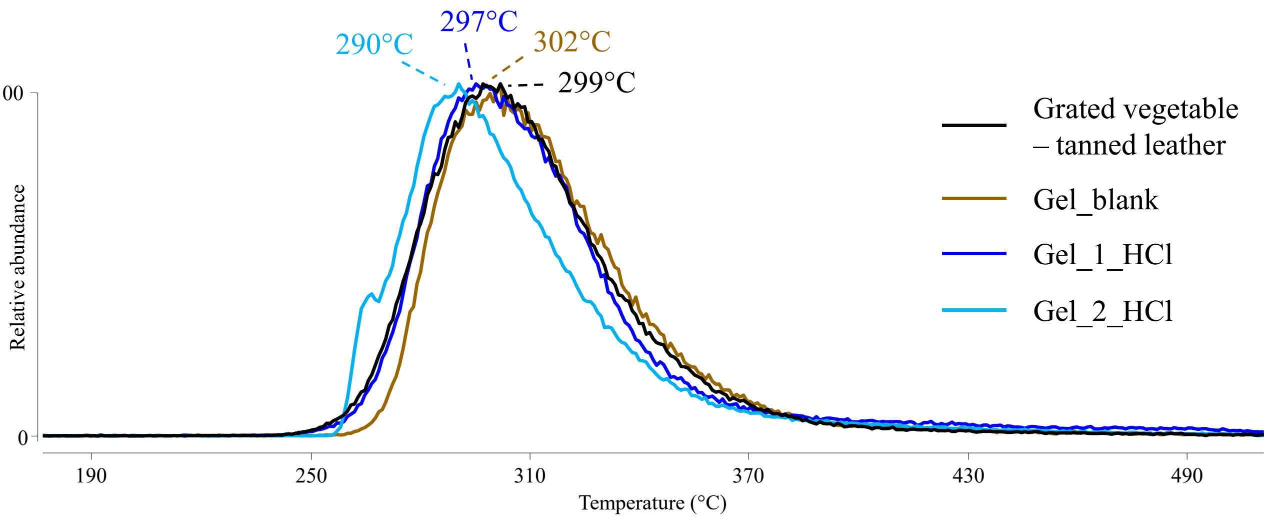

Figure S 9: Extracted Ion Thermograms (EITs) of fragment ion m/z 154 of grated vegetable – tanned leather (black), Gel_blank (brown), Gel_1_HCl (blue) and Gel_2_HCl (light blue).

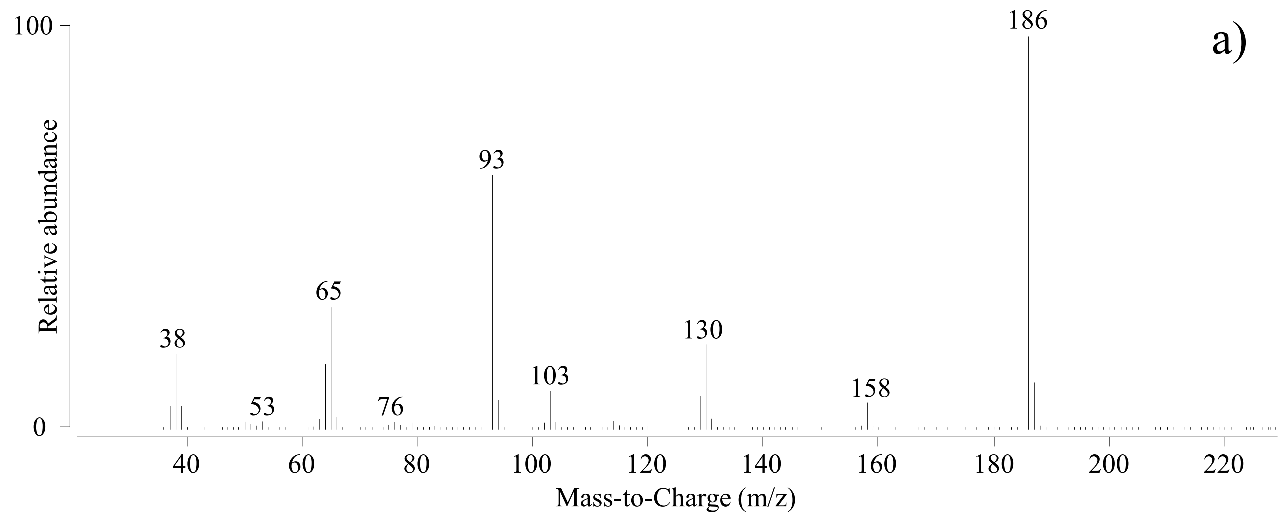

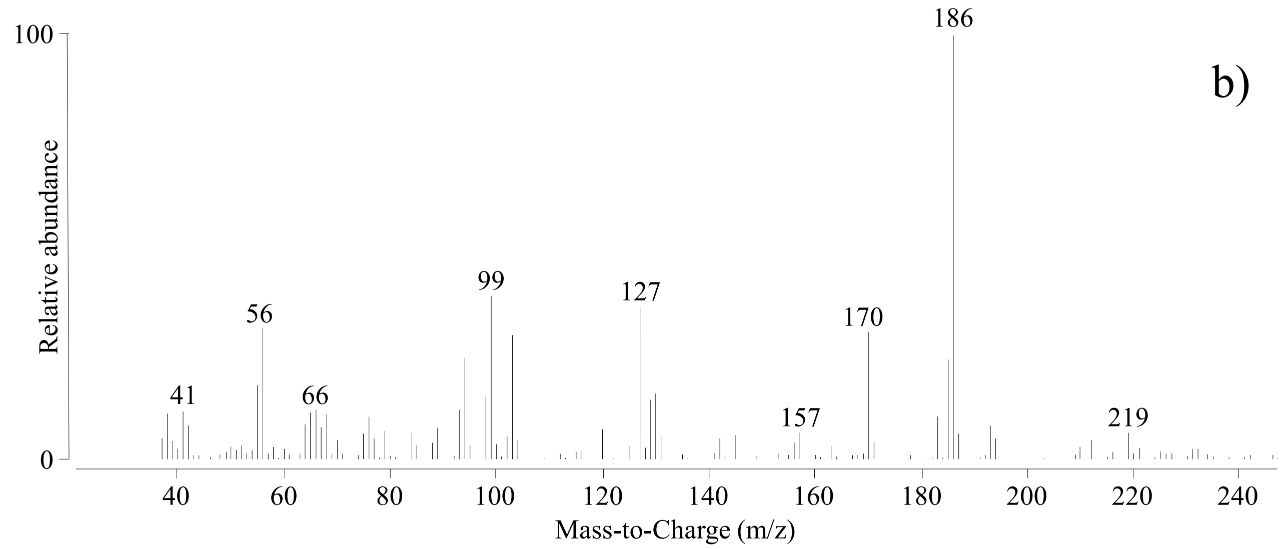

Figure S 10: Mass spectra of heteroaromatic compounds from EICs in Fig 4 at a) 19.1 min and b) 22.9 min.

Table S 6: Summary of protein identification results.

For each protein, data shows number of identified peptides, percentage of sequence coverage, number of peptide isoforms, and counts of unmodified and modified peptides. Modified peptide counts represent the number of peptide isoforms containing the specific modification used for calculating modification extent.

Sample	***Protein name (Uniprot ID)***	***Identified peptides***	***Sequence coverage(%)***	***Peptide isoforms***	***Unmodified Peptides***	***Oxidized M***	***Deamidated N***	***Deamidated Q***
Gel_1_REP1	*Collagen alpha-1(I) chain (P02453)*	*77*	*58.8*	*255*	*31*	*14*	*24*	*33*
Gel_1_REP2		*46*	*42.9*	*145*	*11*	*16*	*16*	*15*
Gel_1_HCl_REP1		*72*	*59.3*	*289*	*30*	*20*	*37*	*53*
Gel_1_HCl_REP2		*57*	*53.4*	*297*	*25*	*14*	*89*	*49*
Gel_1_NaOH_REP1		*74*	*59.1*	*297*	*57*	*14*	*33*	*33*
Gel_1_NaOH_REP2		*68*	*57.3*	*363*	*47*	*14*	*55*	*41*
Gel_2_REP1		*74*	*57.4*	*300*	*42*	*20*	*23*	*35*
Gel_2_REP2		*65*	*54.0*	*227*	*16*	*21*	*17*	*25*
Gel_2_HCl_REP1		*78*	*61.0*	*310*	*50*	*19*	*29*	*48*
Gel_2_HCl_REP2		*80*	*59.3*	*346*	*55*	*18*	*33*	*44*
Gel_2_NaOH_REP1		*76*	*61.2*	*316*	*47*	*20*	*21*	*37*
Gel_2_NaOH_REP2		*75*	*60.4*	*326*	*53*	*21*	*28*	*38*
Gel_blank_REP1		*63*	*56.2*	*256*	*28*	*17*	*26*	*30*
Gel_blank_REP2		*46*	*44.4*	*105*	*14*	*14*	*9*	*15*
Grated_leather_REP1		*52*	*49.9*	*137*	*16*	*17*	*9*	*19*
Grated_leather_REP2		*30*	*31.6*	*52*	*10*	*11*	*6*	*4*
Gel_1_REP1	*Collagen alpha-2(I) chain (P02465)*	*53*	*54.9*	*108*	*8*	*5*	*28*	*7*
Gel_1_REP2		*28*	*33.6*	*56*	*4*	*0*	*13*	*4*
Gel_1_HCl_REP1		*62*	*66.9*	*165*	*9*	*8*	*43*	*14*
Gel_1_HCl_REP2		*51*	*56.0*	*140*	*6*	*7*	*43*	*14*
Gel_1_NaOH_REP1		*55*	*59.1*	*166*	*12*	*9*	*47*	*14*
Gel_1_NaOH_REP2		*54*	*56.6*	*182*	*17*	*8*	*49*	*12*
Gel_2_REP1		*55*	*58.3*	*132*	*10*	*10*	*36*	*7*
Gel_2_REP2		*48*	*51.8*	*109*	*10*	*8*	*33*	*6*
Gel_2_HCl_REP1		*62*	*64.9*	*171*	*14*	*9*	*42*	*13*
Gel_2_HCl_REP2		*64*	*68.9*	*210*	*11*	*12*	*52*	*13*
Gel_2_NaOH_REP1		*66*	*67.8*	*213*	*14*	*8*	*44*	*10*
Gel_2_NaOH_REP2		*63*	*66.7*	*203*	*11*	*12*	*41*	*8*
Gel_blank_REP1		*51*	*57.0*	*144*	*9*	*13*	*45*	*6*
Gel_blank_REP2		*33*	*36.6*	*63*	*4*	*5*	*19*	*5*
Grated_leather_REP1		*43*	*50.6*	*75*	*6*	*5*	*28*	*3*
Grated_leather_REP2		*22*	*26.6*	*33*	*2*	*1*	*7*	*2*
Gel_1_REP1	*Collagen alpha-1(III) chain (P04258)*	*55*	*70.1*	*168*	*7*	*7*	*42*	*21*
Gel_1_REP2		*33*	*50.5*	*70*	*4*	*8*	*21*	*8*
Gel_1_HCl_REP1		*56*	*66.9*	*168*	*6*	*16*	*48*	*20*
Gel_1_HCl_REP2		*53*	*65.9*	*195*	*8*	*13*	*68*	*29*
Gel_1_NaOH_REP1		*56*	*66.9*	*177*	*7*	*17*	*47*	*18*
Gel_1_NaOH_REP2		*57*	*65.7*	*173*	*6*	*14*	*51*	*21*
Gel_2_REP1		*59*	*70.9*	*188*	*3*	*18*	*48*	*25*
Gel_2_REP2		*46*	*61.2*	*125*	*4*	*13*	*37*	*12*
Gel_2_HCl_REP1		*64*	*70.2*	*197*	*7*	*20*	*56*	*18*
Gel_2_HCl_REP2		*59*	*68.6*	*192*	*8*	*24*	*47*	*20*
Gel_2_NaOH_REP1		*56*	*67.2*	*167*	*6*	*19*	*42*	*12*
Gel_2_NaOH_REP2		*59*	*68.9*	*185*	*8*	*17*	*45*	*21*

Gel_blank_REP1	*48*	*62.8*	*150*	*5*	*10*	*43*	*16*
Gel_blank_REP2	*36*	*44.7*	*70*	*3*	*8*	*22*	*6*
Grated_leather_REP1	*37*	*50.1*	*87*	*3*	*11*	*27*	*10*
Grated_leather_REP2	*20*	*26.9*	*35*	*1*	*5*	*9*	*3*

Table S 4: Overall extent of backbone cleavage.
Data shows the mean ratio of semitryptic to total peptides across samples along with its standard error. Semitryptic/TOT ratios indicate the extent of backbone cleavage in samples, where higher ratios reflect increased protein degradation. Total counts of identified tryptic and semitryptic peptides backbone cleavage data is based on are reported for each sample replicate.

Sample	*Experiment*	*Total Tryptic*	*Total Semi-tryptic*	*Semitryptic/TOT (Mean)*	*Std Error*
Gel_1	*Gel_1_REP1*	*199*	*25*	*0.11*	*0.00*
	Gel_1_REP2	*120*	*16*		
Gel_1_HCl	*Gel_1_HCl_REP1*	*454*	*146*	*0.25*	*0.01*
	Gel_1_HCl_REP2	*466*	*164*		
Gel_1_NaOH	*Gel_1_NaOH_REP1*	*480*	*68*	*0.14*	*0.01*
	Gel_1_NaOH_REP2	*470*	*81*		
Gel_2	*Gel_2_REP1*	*317*	*33*	*0.11*	*0.02*
	Gel_2_REP2	*305*	*45*		
Gel_2_HCl	*Gel_2_HCl_REP1*	*477*	*83*	*0.15*	*0.00*
	Gel_2_HCl_REP2	*517*	*86*		
Gel_2_NaOH	*Gel_2_NaOH_REP1*	*486*	*102*	*0.15*	*0.02*
	Gel_2_NaOH_REP2	*515*	*81*		
Gel_blank	*Gel_blank_REP1*	*282*	*22*	*0.07*	*0.00*
	Gel_blank_REP2	*99*	*7*		
Grated_leather	*Grated_leather_REP1*	*62*	*5*	*0.08*	*0.01*
	Grated_leather_REP2	38	4		

Table S 5: Analysis of protein modification extent.
Mean values and standard errors for the extent of methionine (M) oxidation and deamidation (N|Q) are presented by sample, along with the number of detected and modified amino acid sites the data is based on. N|Q represents combined deamidation at both asparagine (N) and glutamine (Q) residues.

	Oxidation (M)				***Deamidation (N|Q)***			
Sample	***Detected M***	***Modified M***	***Fraction of modified/TOT***	***Std Error***	***Detected N|Q***	***Modified N|Q***	***Fraction of modified/TOT***	***Std Error***
Gel_1	*77*	*67*	*0.86*	*0.01*	*1094*	*485*	*0.33*	*0.02*
Gel_1_HCl	*76*	*65*	*0.86*	*0.01*	*1092*	*394*	*0.44*	*0.06*
Gel_1_NaOH	*49*	*42*	*0.86*	*0.03*	*624*	*198*	*0.36*	*0.00*
Gel_2	*99*	*90*	*0.89*	*0.04*	*1168*	*403*	*0.31*	*0.00*
Gel_2_HCl	*106*	*92*	*0.90*	*0.00*	*1085*	*334*	*0.34*	*0.00*
Gel_2_NaOH	*70*	*63*	*0.87*	*0.03*	*747*	*229*	*0.31*	*0.02*
Gel_blank	*58*	*48*	*0.85*	*0.10*	*509*	*177*	*0.37*	*0.05*
Grated_leather	*35*	*31*	*0.90*	*0.00*	*236*	*96*	*0.40*	*0.00*

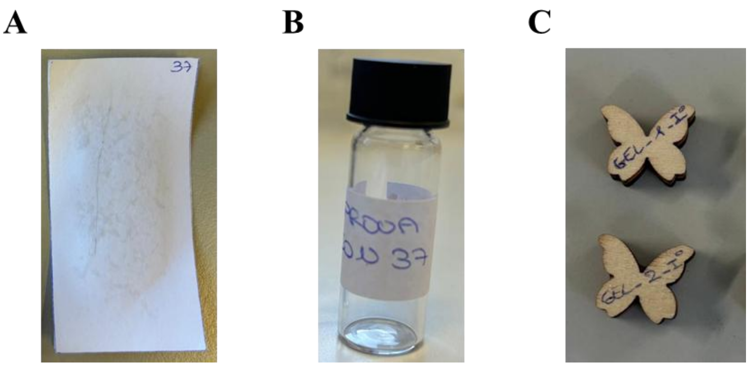

Figure S 11: Adhesion tests with Gel_1 as bioadhesive. A) paper–paper, B) paper–glass, and C) wood–wood.

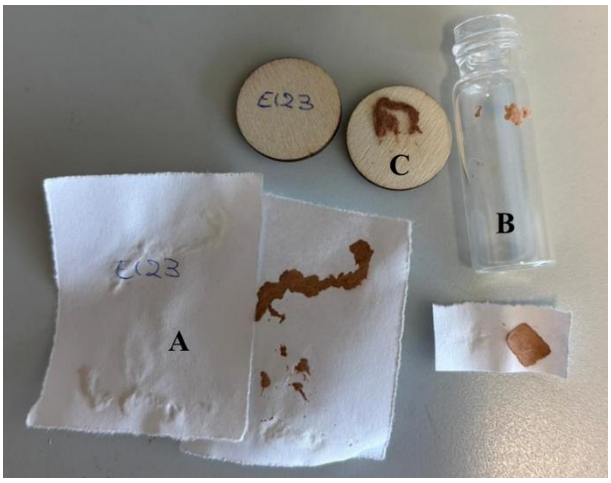

Figure S 12: Adhesion test with control experiment (Gel-blank). A) paper–paper, B) paper–glass, and C) wood–wood.

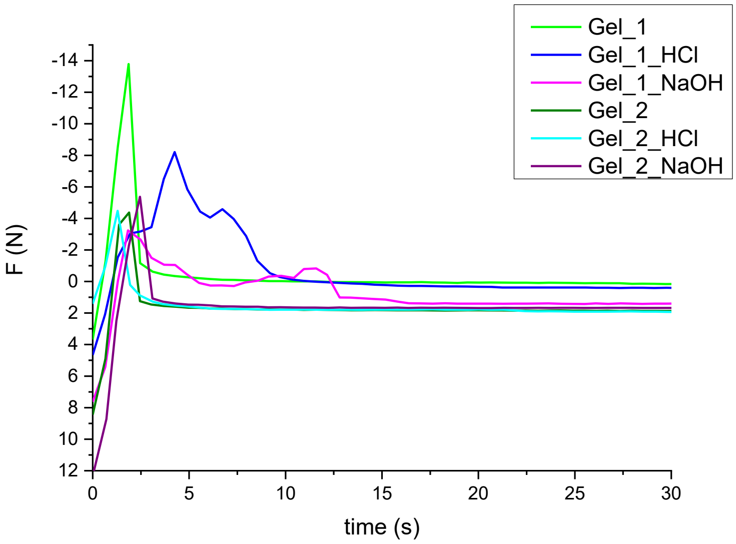

Figure S 13: tack test made on the samples to test the adhesive strength on steel-wood surfaces.

Table S 6: Scores attributed to each principle to evaluate the process greenness, made according to Path2Green application.

Principles		Score*	Motivation	Weight**
**1**	**Biomass**	+1	Waste biomasses	6
**2**	**Transport**	+0.5	40 km distance	5
**3**	**Pre-treatment**	-0.2	Physical pretreatment	2.5
**4**	**Solvent**	+1	Recommended solvents (DESs)	6
**5**	**Scaling**	-1	In batches	3
**6**	**Purification**	-0.5	Water	2.5
**7**	**Yield**	+1	Complete valorization of the biomass	4
**8**	**Post-treat.**	+1	Ready-to-use extract	2.5
**9**	**Energy**	+0.5	Low energy dependence	5
**10**	**Application**	+0.66	To be applied in four domains	4.5
**11**	**Repurposing**	+1	Non-Virgin raw materials are used	6
**12**	**Waste**	-1	Waste generated: 100% m/m	6

**Scores and weights were chosen following the recommendations of (DOI: 10.1039/d4gc02512a).*
